# Medicaid Expansion and Medicare-Financed Hospitalizations Among Adult Patients With Incident Kidney Failure

**DOI:** 10.1001/jamahealthforum.2022.3878

**Published:** 2022-11-04

**Authors:** Kevin H. Nguyen, Yoojin Lee, Rebecca Thorsness, Maricruz Rivera-Hernandez, Daeho Kim, Shailender Swaminathan, Rajnish Mehrotra, Amal N. Trivedi

**Affiliations:** 1 Department of Health Services, Policy, and Practice, Brown University School of Public Health, Providence, Rhode Island; 2 Chief Medical Office, Veterans Affairs New England Healthcare System, Bedford, Massachusetts; 3 Sapien Labs Centre for Human Brain and Mind, Krea University, India; 4 Department of Medicine, University of Washington School of Medicine, Seattle; 5 Providence Veterans Affairs Medical Center, Providence, Rhode Island

## Abstract

**Importance:**

Although Medicare provides health insurance coverage for most patients with kidney failure in the US, Medicare beneficiaries who initiate dialysis without supplemental coverage are exposed to substantial out-of-pocket costs. The availability of expanded Medicaid coverage under the Patient Protection and Affordable Care Act (ACA) for adults with kidney failure may improve access to care and reduce Medicare-financed hospitalizations after dialysis initiation.

**Objective:**

To examine the implications of the ACA’s Medicaid expansion for Medicare-financed hospitalizations, health insurance coverage, and predialysis nephrology care among Medicare-covered adults aged 19 to 64 years with incident kidney failure in the first year after initiating dialysis.

**Design, Setting, and Participants:**

This cross-sectional study used a difference-in-differences approach to assess Medicare-financed hospitalizations among adults aged 19 to 64 years who initiated dialysis between January 1, 2010, and December 31, 2018, while covered by Medicare Part A (up to 5 years postexpansion). Data on patients were obtained from the Renal Management Information System’s End Stage Renal Disease Medical Evidence Report, which includes data for all patients initiating outpatient maintenance dialysis regardless of health insurance coverage, treatment modality, or citizenship status, and these data were linked with claims data from the Medicare Provider Analysis and Review. Data were analyzed from January to August 2022.

**Exposure:**

Living in a Medicaid expansion state.

**Main Outcomes and Measures:**

Primary outcomes were number of Medicare-financed hospitalizations and hospital days in the first 3 months, 6 months, and 12 months after dialysis initiation. Secondary outcomes included dual Medicare and Medicaid coverage at 91 days after dialysis initiation and the presence of an arteriovenous fistula or graft at dialysis initiation for patients undergoing hemodialysis.

**Results:**

The study population included 188 671 adults, with 97 071 living in Medicaid expansion states (mean [SD] age, 53.4 [9.4] years; 58 329 men [60.1%]) and 91 600 living in nonexpansion states (mean [SD] age, 53.0 [9.6] years; 52 677 men [57.5%]). In the first 3 months after dialysis initiation, Medicaid expansion was associated with a significant decrease in Medicare-financed hospitalizations (−4.24 [95% CI, −6.70 to −1.78] admissions per 100 patient-years; *P* = .001) and hospital days (−0.73 [95% CI, −1.08 to −0.39] days per patient-year; *P* < .001), relative reductions of 8% for both outcomes. Medicaid expansion was associated with a 2.58–percentage point (95% CI, 0.88-4.28 percentage points; *P* = .004) increase in dual Medicare and Medicaid coverage at 91 days after dialysis initiation and a 1.65–percentage point (95% CI, 0.31-3.00 percentage points; *P* = .02) increase in arteriovenous fistula or graft at initiation.

**Conclusions and Relevance:**

In this cross-sectional study with a difference-in-differences analysis, the ACA’s Medicaid expansion was associated with decreases in Medicare-financed hospitalizations and hospital days and increases in dual Medicare and Medicaid coverage. These findings suggest favorable spillover outcomes of Medicaid expansion to Medicare-financed care, which is the primary payer for patients with kidney failure.

## Key Points

**Question:**

Was Medicaid expansion under the Patient Protection and Affordable Care Act (ACA) associated with lower rates of Medicare-financed hospitalizations among adults with incident kidney failure?

**Findings:**

In this cross-sectional study of 188 671 US adults aged 19 to 64 years with kidney failure initiating dialysis, the ACA Medicaid expansion was associated with a reduction in the number of Medicare-financed hospitalizations in the first 3 months after dialysis initiation.

**Meaning:**

Findings of this study suggest that decreases in Medicare-financed hospitalizations may indicate favorable spillover outcomes from the ACA’s Medicaid expansion to Medicare through reduced spending on hospitalizations among a clinically complex patient population.

## Teaser

This cross-sectional study assesses whether the Medicaid expansion implemented as part of the Patient Protection and Affordable Care Act was associated with changes in Medicare-financed hospitalizations for adults with incident kidney failure.

## Introduction

Nearly 800 000 people in the US have kidney failure, with Black and Hispanic or Latino populations and those with low household incomes disproportionately affected.^1,2,3^ Among persons with kidney failure treated with dialysis, the period immediately after initiating dialysis carries substantial risk of mortality, frequent hospitalizations, infections, and cardiovascular events.^4,5,6^ Although mortality rates have declined in recent years, reported mortality rates among patients with incident kidney failure were 8% at 90 days and 22% at 1 year.^7^

Medicare is the primary insurer for individuals in the US aged 65 years and older as well as those younger than 65 with disabilities who receive Social Security Disability Insurance (SSDI). Medicare also provides health insurance coverage for most patients with kidney failure, and coverage begins at 91 days after initiating in-center hemodialysis or at the time of enrolling in training for home-based dialysis. In 2017, Medicare fee-for-service spending for all beneficiaries with kidney failure was approximately $36 billion (or 6% of total spending).^1^ However, Medicare beneficiaries who initiate dialysis without supplemental coverage (eg, Medigap plans, employer-sponsored retiree benefits, or Medicaid) are exposed to substantial out-of-pocket costs, and Medicare Part D prescription drug coverage requires premiums and cost sharing.^8,9^ As part of the Patient Protection and Affordable Care Act (ACA), states had the option of expanding Medicaid eligibility to adults with low household incomes, and as of April 2022, 38 states and the District of Columbia have done so.^10^ Medicaid expansion has had favorable outcomes for patients with kidney disease, including increases in health insurance coverage, Medicaid-covered preemptive listings for kidney transplantation, and 1-year survival after dialysis initiation.^11,12^ State Medicaid expansion decisions and Medicaid generosity (eg, levels of state Medicaid coverage) are associated with lower kidney failure incidence and increased use of an arteriovenous fistula or graft at dialysis initiation.^13,14^

In addition to providing assistance with Medicare premiums and cost sharing,^15^ Medicaid expansion may facilitate access to predialysis nephrology care for adult patients initiating dialysis,^8^ thereby preventing complications and reducing the number of Medicare-financed hospitalizations and hospital days immediately after dialysis initiation. These implications may be evident among adults with low household incomes eligible for Medicaid after the ACA and for those who were previously eligible for Medicaid but enrolled in the program after the ACA expansions, a phenomenon sometimes referred to as the woodwork or welcome mat effect. The aim of the study was to examine the implications of Medicaid expansion for Medicare-financed hospitalizations, health insurance coverage, and receipt of predialysis nephrology care among Medicare-covered adults aged 19 to 64 years with incident kidney failure.

## Methods

### Study Design

In this cross-sectional study, we used a difference-in-differences approach to compare changes in the number of Medicare-financed hospitalizations, health insurance coverage, and receipt of predialysis nephrology care over time in Medicaid expansion vs nonexpansion states. The study population included persons aged 19 to 64 years with kidney failure who initiated dialysis between January 1, 2010, and December 31, 2018, while covered by Medicare Part A. The study period included 4 years during which patients initiated treatment before Medicaid expansion (2010-2013) and 5 years after expansion (2014-2018). Consistent with previous work, we considered expansion states as those that implemented the ACA’s Medicaid expansion from 2014 and afterward and excluded 5 states that extended Medicaid eligibility to adults with low household incomes between 2010 and 2013 (eTable 1 in the Supplement).^16,17^ Each state’s postexpansion period was defined by its own implementation date, which was January 1, 2014, for most states. The Brown University Institutional Review Board and the Centers for Medicare & Medicaid Services (CMS) Privacy Board approved the study protocol and waived the requirement for informed consent because only deidentified data were used. The study followed the Strengthening the Reporting of Observational Studies in Epidemiology (STROBE) reporting guideline for cross-sectional studies.

### Data Sources

We used data from the Renal Management Information System’s End Stage Renal Disease Medical Evidence Report (CMS 2728), which is completed for all people initiating outpatient maintenance dialysis regardless of health insurance coverage, treatment modality, or citizenship status.^18^ Because CMS 2728 includes patients’ primary mailing addresses, we geolocated patients into US Census tracts using ArcGIS spatial mapping software, version 10.5.1 (Esri).^3,11,13,19^ Hospitalizations were assessed by linking the CMS 2728 data to the Medicare Provider Analysis and Review, which includes information about all Medicare-financed hospitalizations, including primary diagnoses and number of hospital days. Data sets were linked using patients’ Medicare beneficiary identifiers. We included patients with both traditional Medicare and Medicare Advantage because the Medicare Provider Analysis and Review includes more than 90% of hospitalizations for enrollees in Medicare Advantage.^20,21^ The 2009 to 2013 American Community Survey data provided the poverty rate in each patient’s US Census tract.

### Outcomes

The primary outcomes were the number of Medicare-financed acute care hospitalizations and number of acute care hospital days in the first 3 months, 6 months, and 12 months after initiation of dialysis. Secondary outcomes were dual Medicare and Medicaid coverage at 91 days after dialysis initiation, receipt of predialysis nephrology care, presence of arteriovenous fistula (AVF) or graft at dialysis initiation for patients undergoing hemodialysis, receipt of home dialysis, and dialysis type at initiation (hemodialysis vs peritoneal dialysis). We also assessed hospitalizations due to cardiovascular disease or infectious conditions using the US Renal Data System’s approach to classify these conditions.^22^ We attributed hospitalizations to the quarter of the patient’s date of dialysis initiation.

### Statistical Analysis

Data were analyzed from January to August 2022. We used a linear regression model with Huber-White robust SEs clustered at the state level. Covariates included age, sex, race and ethnicity, primary cause of kidney failure, presence of congestive heart failure, atherosclerotic heart disease, other cardiac disease, hypertension, diabetes, diabetic retinopathy, cancer, obesity (body mass index >30, calculated as weight in kilograms divided by height in meters squared), smoking status, alcohol dependence, and hemoglobin and serum albumin levels at dialysis initiation.^11^ The CMS 2728 specifies that a patient’s race and ethnicity should be collected using patient self-report at treatment initiation and was classified as Hispanic or Latino, non-Hispanic African American or Black, non-Hispanic Asian, non-Hispanic White, or non-Hispanic other race (American Indian or Alaska Native, Native Hawaiian or Pacific Islander, or other race). Consistent with previous work, for observations missing serum albumin and hemoglobin levels, we used the mean value of the covariates for nonmissing observations.^11^ All models included state and year-quarter fixed effects. Analyses were conducted in Stata, version 17 (StataCorp LLC) and used 2-tailed hypothesis testing with a significance threshold of *P* < .05.

We compared characteristics of persons with kidney failure aged 19 to 64 years who had Medicare Part A at treatment initiation vs those who did not during the study period to assess the generalizability of our findings. To assess the validity of the difference-in-differences study design and to test the robustness of the findings, we conducted several sensitivity analyses (eAppendix in the Supplement). First, we visually inspected preexpansion trends. Using quarterly data before 2014, we then tested the statistical significance of an expansion-by-time trend and separately used a categorical time specification. Second, we reran the analyses to include states that expanded Medicaid before January 1, 2014, or late-expanding states (eTable 1 in the Supplement). Third, we examined changes in patient characteristics over time by state expansion status to account for potential shifts in patient composition. Fourth, we used a Poisson model to examine changes in number of hospital days. Fifth, we ascertained the sensitivity of the results to inclusion and exclusion of hemoglobin and serum albumin levels in the risk-adjusted model and included missing hemoglobin and serum albumin levels as an indicator variable. Sixth, we modeled the postperiod as an event study (comparing annual changes in outcomes to a pooled preperiod), for which a state’s postexpansion period was defined by its own implementation date (eTable 1 in the Supplement). Seventh, in exploratory analyses, we examined whether there were differential changes in outcomes by age, sex, race and ethnicity, or area-level poverty (ie, living in a US Census tract where 20% or more of the population was living below the poverty threshold, which varies based on the size of the family and number of children in the household) by testing the significance of 3-way interactions among expansion status, time period, and each characteristic. In addition, to account for the competing risk of death, we calculated mortality rates within 3-month, 6-month, and 12-month periods after initiating treatment.

## Results

The study population included 188 671 adults aged 19 to 64 years who initiated dialysis while covered by Medicare Part A. Of this total, 97 071 resided in Medicaid expansion states (mean [SD] age, 53.4 [9.4] years; 58 329 men [60.1%] and 38 742 women [39.9%]; 13.3% Hispanic or Latino, 26.4% non-Hispanic African American or Black, 3.4% non-Hispanic Asian, and 54.5% non-Hispanic White individuals and 2.5% non-Hispanic individuals of other races), and 91 600 resided in nonexpansion states (mean [SD] age, 53.0 [9.6] years; 52 677 men [57.5%] and 38 923 women [42.5%]; 12.6% Hispanic or Latino, 40.2% non-Hispanic African American or Black, 1.0% non-Hispanic Asian, and 45.0% non-Hispanic White individuals and 1.2% non-Hispanic individuals of other races) (Table 1). The most common original reason for Medicare eligibility among both groups was disability insurance benefits (59.5% in expansion states, 51.9% in nonexpansion states) and disability and end-stage kidney disease (24.5% in expansion states, 29.7% in nonexpansion states).

**Table 1.** Characteristics of Adults Aged 19 to 64 Years With Medicare Part A Coverage at Dialysis Initiation by State Medicaid Expansion Status, 2010 to 2018^a^^,^^b^

Characteristic	Patients, No. (%)		*P* value
	Residing in expansion states (n = 97 071)	Residing in nonexpansion states (n = 91 600)	
Age, mean (SD), y	53.4 (9.4)	53.0 (9.6)	<.001
Age category, y			
19-34	5442 (5.6)	5516 (6.0)	<.001
35-44	10 739 (11.1)	11 114 (12.1)	
45-54	26 075 (26.9)	25 171 (27.5)	
55-64	54 815 (56.5)	49 799 (54.4)	
Sex			
Male	58 329 (60.1)	52 677 (57.5)	<.001
Female	38 742 (39.9)	38 923 (42.5)	
Race and ethnicity			
Hispanic or Latino	12 925 (13.3)	11 507 (12.6)	<.001
Non-Hispanic African American or Black	25 585 (26.4)	36 787 (40.2)	
Non-Hispanic Asian	3276 (3.4)	946 (1.0)	
Non-Hispanic White	52 902 (54.5)	41 217 (45.0)	
Non-Hispanic other race^c^	2383 (2.5)	1143 (1.2)	
Original reason for Medicare entitlement			
Old age and survivor’s insurance	460 (0.5)	407 (0.4)	.34
Disability insurance benefits	57 784 (59.5)	47 572 (51.9)	<.001
End-stage kidney disease	15 037 (15.5)	16 429 (17.9)	<.001
Disability and end-stage kidney disease	23 790 (24.5)	27 192 (29.7)	<.001
Primary cause of kidney failure^d^			
Diabetes	47 398 (48.8)	44 327 (48.4)	.06
Hypertension	18 300 (18.9)	21 521 (23.5)	<.001
Other	31 367 (32.3)	25 740 (28.1)	<.001
Comorbid conditions			
Congestive heart failure	25 101 (25.9)	23 172 (25.3)	.005
Atherosclerotic heart disease	12 111 (12.5)	10 572 (11.5)	<.001
Other cardiac disease	15 952 (16.4)	14 734 (16.1)	.04
Hypertension	84 366 (86.9)	81 511 (89.0)	<.001
Diabetes	60 257 (62.1)	57 715 (63.0)	<.001
Diabetic retinopathy	9895 (10.2)	9006 (9.8)	.009
Cancer	4366 (4.5)	3759 (4.1)	
BMI >30 (obesity)^e^	46 654 (48.1)	45 652 (49.8)	<.001
Current smoker	8476 (8.7)	8666 (9.5)	<.001
Alcohol dependence	1792 (1.8)	1492 (1.6)	<.001
Hemoglobin level, mean (SD), g/dl^f^	9.65 (7.91)	9.69 (14.65)	.49
Serum albumin level, mean (SD), g/dl^g^	3.22 (2.48)	3.19 (3.32)	.03
% Of patients in US Census tract living below poverty threshold, mean (SD)^h^	20.06 (11.02)	22.12 (10.52)	<.001

Abbreviation: BMI, body mass index (calculated as weight in kilograms divided by height in meters squared).

SI conversion factors: To convert albumin level to grams per liter, multiply by 10; to convert hemoglobin level to grams per liter, multiply by 10.

^a^ Sample deduplicated patients with multiple events because there were 190 378 events for 188 671 patients, and the first observation in the End Stage Renal Disease Medical Evidence Report was used.

^b^ Individuals initiated dialysis between January 1, 2010, and December 31, 2018.

^c^ Other races included American Indian or Alaska Native, Native Hawaiian or Other Pacific Islander, and those who marked “other” race.

^d^ The primary cause of kidney failure was missing for less than 1% of patients residing in expansion (n = 6) and nonexpansion states (n = 12). Other primary causes include, for example, glomerulonephritis, interstitial nephritis or pyelonephritis, and transplant complications.

^e^ Obesity data were missing for less than 1% of patients residing in expansion (n = 381) and nonexpansion states (n = 356).

^f^ Hemoglobin levels were missing for 15.0% of patients residing in expansion states (n = 14 531) and 18.7% of patients residing in nonexpansion states (n = 17 148).

^g^ Serum albumin levels were missing for 32.4% of patients residing in expansion states (n = 31 453) and 30.6% of patients residing in nonexpansion states (n = 28 050).

^h^ Area-level poverty refers to the US Census tract–level proportion of the population living below the poverty threshold, which varies based on the size of the family and number of children in the household.

### Primary and Secondary Outcomes

**Figure 1.**
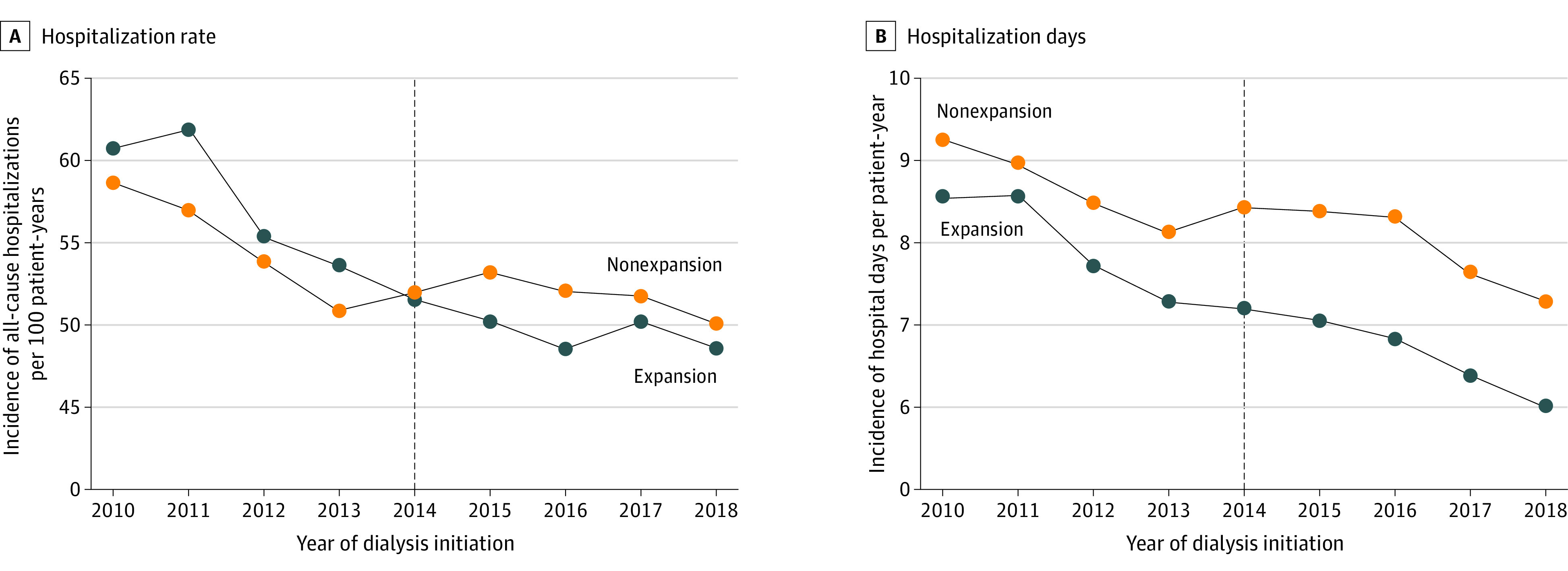
Changes in All-Cause Hospitalizations and Hospital Days Within 3 Months of Dialysis Initiation Expansion states were limited to those that expanded Medicaid coverage in 2014 and exclude those that did not expand in 2015 and afterward. The vertical line represents Medicaid expansion in 2014.

In the first 3 months after dialysis initiation, Medicaid expansion was associated with a significant decrease in Medicare-financed acute care hospitalizations (−4.24 [95% CI, −6.70 to −1.78] admissions per 100 patient-years; *P* = .001) and acute care hospital days (−0.73 [95% CI, −1.08 to −0.39] days per patient-year; *P* < .001), with relative reductions of 8% (Table 2, Figure 1). Medicaid expansion was also associated with significant decreases in hospitalizations (−5.79 [95% CI, −10.36 to −1.23] admissions per 100 patient-years; *P* = .01) in the first 6 months after initiation of dialysis (eFigures 1 and 2 in the Supplement).

**Table 2.** Changes in Medicare and Medicaid Coverage, Predialysis Nephrology Care, All-Cause Hospitalization Rates, and Number of Hospital Days Among Adults Aged 19 to 64 Years Initiating Dialysis After Medicaid Expansion^a^

Outcome	Expansion states			Nonexpansion states			Adjusted difference-in-differences estimate (95% CI)	*P* value
	Baseline	Postexpansion	Change	Baseline	Postexpansion	Change		
**Primary outcomes**								
No. of hospital admissions per 100 patient-years								
3 mo After dialysis initiation	58.43	51.05	−7.38	55.01	51.85	−3.16	−4.24 (−6.70 to −1.78)	.001
6 mo After dialysis initiation	103.80	92.12	−11.67	98.33	92.32	−6.00	−5.79 (−10.36 to −1.23)	.01
12 mo After dialysis initiation	182.20	165.87	−16.33	171.87	164.76	−7.11	−9.27 (−17.81 to −0.73)	.03
No. of hospital days per patient-year								
3 mo After dialysis initiation	8.46	6.94	−1.52	8.68	8.02	−0.66	−0.73 (−1.08 to −0.39)	<.001
**Secondary outcomes**								
Dual Medicare and Medicaid coverage	45.30	48.96	3.66	42.53	43.94	1.41	2.58 (0.88 to 4.28)	.004
Receipt of predialysis nephrology care^b^	76.35	81.54	5.19	75.09	79.35	4.26	0.93 (−0.08 to 1.95)	.07
Arteriovenous fistula or graft^c^	23.84	24.68	0.84	22.76	22.21	−0.55	1.65 (0.31 to 3.00)	.02
Home dialysis	16.80	18.47	1.67	19.42	21.10	1.68	0.05 (−1.44 to 1.54)	.95
Type of dialysis at initiation								
Hemodialysis	83.99	82.31	−1.68	81.60	79.86	−1.74	−0.03 (−0.14 to 1.31)	.97
Peritoneal dialysis	16.01	17.69	1.68	18.40	20.14	1.74	0.03 (−1.31 to 0.14)	.97

^a^ Point estimates at baseline and postexpansion are unadjusted. Adjusted models included indicators for Medicaid expansion, the postperiod, and their interaction (expansion × postperiod). A state’s postperiod was defined by its own implementation date, which was January 1, 2014, for most expansion states. Models were also adjusted for age, sex, race and ethnicity, primary cause of kidney failure, comorbid conditions, current smoker status, alcohol dependence, body mass index, hemoglobin and serum albumin levels at dialysis initiation, and area-level poverty. The model included quarter-year and state fixed effects, and SEs were clustered at the state level.

^b^ This variable was unknown for 12.0% of patients.

^c^ This variable was unknown for 18.2% of patients and included only patients undergoing dialysis. Estimates for number of hospital days per patient-year 6 and 12 months after dialysis initiation were excluded because trends between Medicaid expansion and nonexpansion states were not parallel.

**Figure 2.**
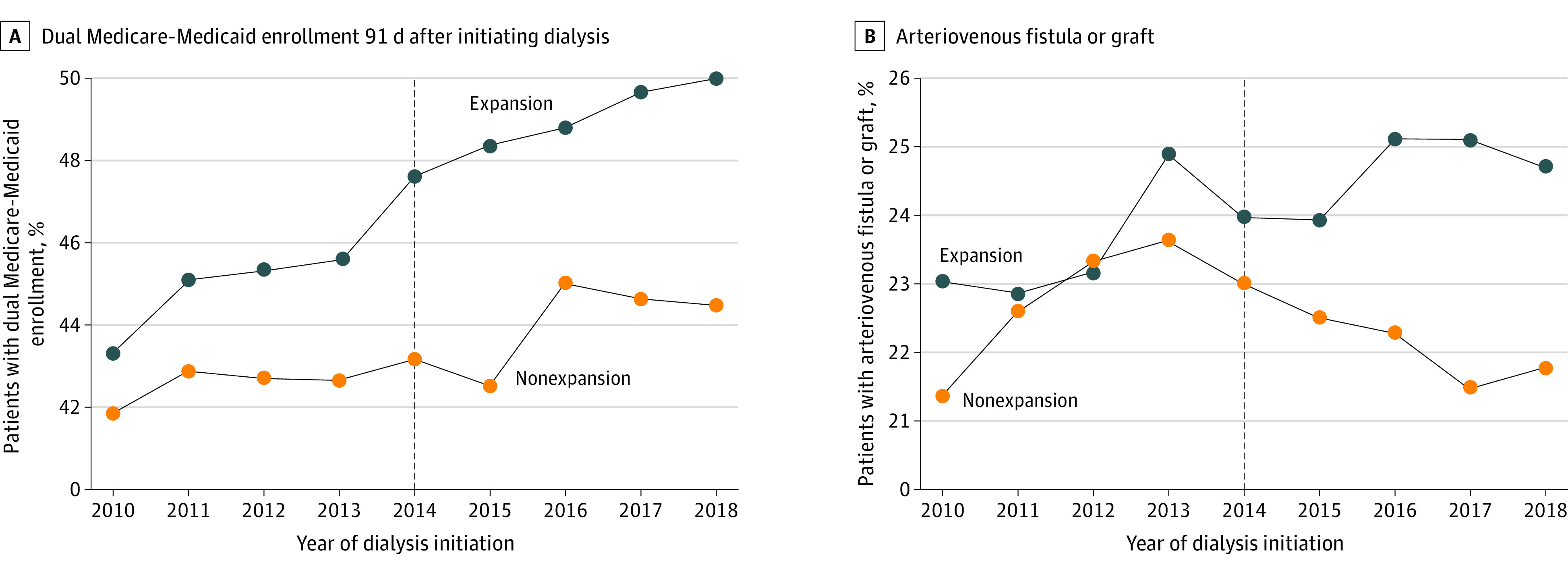
Changes in Dual Eligible Enrollment 91 Days After Initiating Dialysis, by Medicaid Expansion State, and Development of an Arteriovenous Fistula or Graft Expansion states were limited to those that expanded Medicaid coverage in 2014 and excludes those that did not expand in 2015 and afterward. The vertical line represents Medicaid expansion in 2014.

Medicaid expansion was associated with a 2.58–percentage point increase (95% CI, 0.88-4.28 percentage points; *P* = .004) in dual Medicare and Medicaid coverage at 91 days after dialysis initiation (a 6% relative increase) (Table 2, Figure 2A). Although there were no statistically significant differences in receipt of predialysis nephrology care, home dialysis, or dialysis type (hemodialysis or peritoneal) by state expansion status (eFigures 3 and 4 in the Supplement), Medicaid expansion was associated with a significant 1.65–percentage point increase (95% CI, 0.31-3.00 percentage points; *P* = .02) in the presence of an AVF or graft at dialysis initiation for patients undergoing hemodialysis (a 7% relative increase) (Figure 2B).

Medicaid expansion was associated with a significant reduction in the number of hospital days for cardiac conditions in the 3 months after dialysis initiation (−0.13 [95% CI, −0.24 to −0.01] days per patient-year; *P* = .04) (Table 3; eFigures 5-7 in the Supplement). Medicaid expansion was also associated with significant decreases in the number of hospitalizations related to infections at 3 months (−1.55 [95% CI, −2.41 to −0.68] admissions per 100 patient-years; *P* < .001), 6 months (−1.76 [95% CI, −3.06 to −0.46] admissions per 100 patient-years; *P* = .009), and 12 months (−3.23 [95% CI, −5.41 to −1.06] admissions per 100 patient-years; *P* = .004) after dialysis initiation (Table 3; eFigures 8-10 in the Supplement). Medicaid expansion was also associated with fewer hospital days for infection-related hospitalizations 3 months (−0.22 [95% CI, −0.34 to −0.09] days per patient-year; *P* = .001) and 6 months (−0.22 [95% CI, −0.40 to −0.04] days per patient-year; *P* = .02) after dialysis initiation.

**Table 3.** Changes in Cardiac and Infection-Related Hospitalization Rates and Number of Hospital Days Among Adults Aged 19 to 64 Years Initiating Dialysis After Medicaid Expansion^a^

	Expansion states			Nonexpansion states			Adjusted difference-in-differences estimate (95% CI)	*P* value
	Baseline	Postexpansion	Change	Baseline	Postexpansion	Change		
**Cardiac hospitalizations**								
No. of hospital admissions per 100 patient-years								
3 mo After dialysis initiation	14.95	13.26	−1.68	15.43	14.49	−0.94	−0.58 (−1.63 to 0.46)	.27
6 mo After dialysis initiation	25.64	22.56	−3.08	25.94	24.23	−1.71	−1.22 (−2.81 to 0.36)	.13
12 mo After dialysis initiation	45.34	40.07	−5.27	45.15	41.87	−3.28	−1.80 (−4.66 to 1.07)	.21
No. of hospital days per patient-year								
3 mo After dialysis initiation	1.92	1.70	−0.22	2.20	2.14	−0.06	−0.13 (−0.24 to −0.01)	.04
6 mo After dialysis initiation	2.61	2.32	−0.29	2.90	2.79	−0.10	−0.16 (−0.33 to 0.01)	.07
12 mo After dialysis initiation	3.82	3.44	−0.39	4.13	3.95	−0.18	−0.18 (−0.41 to 0.05)	.12
**Infection-related hospitalizations**								
No. of hospital admissions per 100 patient-years								
3 mo After dialysis initiation	15.30	13.67	−1.63	13.70	13.63	−0.07	−1.55 (−2.41 to −0.68)	<.001
6 mo After dialysis initiation	28.41	26.09	−2.32	25.96	25.42	−0.54	−1.76 (−3.06 to −0.46)	.009
12 mo After dialysis initiation	49.43	45.83	−3.60	45.18	44.85	−0.33	−3.23 (−5.41 to −1.06)	.004
No. of hospital days per patient-year								
3 mo After dialysis initiation	2.08	1.81	−0.27	2.01	1.99	−0.02	−0.22 (−0.34 to −0.09)	.001
6 mo After dialysis initiation	3.36	3.03	−0.33	3.29	3.21	−0.08	−0.22 (−0.40 to −0.04)	.02
12 mo after dialysis initiation	5.37	4.88	−0.49	5.22	5.11	−0.12	−0.31 (−0.63 to 0.01)	.06

^a^ Point estimates at baseline and postexpansion are unadjusted. Adjusted models included indicators for Medicaid expansion, the postperiod, and their interaction (expansion × postperiod). A state’s postperiod was defined by its own implementation date, which was January 1, 2014, for most expansion states. Models were also adjusted for age, sex, race and ethnicity, primary cause of kidney failure, comorbid conditions, being a current smoker, alcohol dependence, body mass index, hemoglobin and serum albumin levels at dialysis initiation, and area-level poverty. The model included quarter-year and state fixed effects, and SEs were clustered at the state level.

### Sensitivity Analysis

A total of 212 221 persons (37.8%) aged 19 to 64 years with kidney failure who initiated dialysis had Medicare Part A coverage (eTable 2 in the Supplement). There were several differences in sociodemographic and clinical characteristics between adult persons aged 19 to 64 years with kidney failure who initiated dialysis and had Medicare Part A coverage compared with those who did not have such coverage. Before 2014, trends in outcomes were not statistically different by state expansion status for all outcomes except number of all-cause hospital days per patient-year within 6 months and 12 months of dialysis initiation. We therefore did not investigate the implications of Medicaid expansion for these outcomes (eTable 3, eFigures 11-16 in the Supplement). There were some changes in the characteristics of persons with kidney failure over time in both expansion and nonexpansion states, but these changes were modest (eTable 4 in the Supplement). Estimates were robust to different model specifications, although magnitudes of differences were attenuated when we included states that expanded Medicaid before 2014 (eTable 5 in the Supplement). Estimates using Poisson models were similar in direction and statistical significance, although the magnitude of differences was smaller compared with the main model. There was some variation in changes over time when respecifying the postperiod as an event study (eTable 6 in the Supplement). In exploratory analyses of differential outcomes by patient sociodemographic characteristics, we observed only statistically larger increases in dual Medicare and Medicaid coverage at 91 days after dialysis initiation among Hispanic or Latino patients (change by expansion status, 7.40 percentage points) compared with White patients (change by expansion status, 1.81 percentage points; 3-way interaction, 5.59 [95% CI, 3.32-7.86] percentage points; *P* < .001) (eTables 7-15 in the Supplement). We did not identify statistically significant changes in 3- and 6-month mortality rates by state expansion status; however, Medicaid expansion was associated with a significantly lower 12-month mortality rate (0.55 [95% CI, −1.09 to −0.01] deaths per 100 patient-years; *P* = .04) (eTable 16 in the Supplement).

## Discussion

Among adults aged 19 to 64 years with kidney failure and Medicare coverage who initiated dialysis, Medicaid expansion was associated with a decrease in Medicare-financed hospitalizations 3 months after dialysis initiation and decreases in the number of hospital days at 3 and 6 months after dialysis initiation. Medicaid expansion was also associated with increases in dual Medicare and Medicaid health insurance coverage, particularly for Hispanic or Latino patients with kidney failure. Although there were no significant changes in the receipt of predialysis nephrology care, rates of dialysis initiation with an AVF or graft present among patients undergoing hemodialysis significantly increased in states that expanded Medicaid. Building on previous work, findings of the present study suggest that, among Medicare beneficiaries aged 19 to 64 years with incident kidney failure, Medicaid expansion was associated with a significant decrease in mortality rates 12 months after dialysis initiation.^11^

This study builds on the previous literature in 3 ways: first, it provides new information about the implications of Medicaid expansion for health insurance coverage gains among those already eligible preexpansion. Previous studies indicated that Medicaid expansion was associated with increases in Medicaid coverage among patients with incident kidney failure.^11,13^ The present study sample was composed of people who had Medicare coverage at dialysis initiation, most of whom were eligible for Medicare because of disability through SSDI. In most states before implementation of the ACA, SSDI Medicare beneficiaries aged 19 to 64 years were eligible only for supplemental Medicaid coverage if they concurrently received Supplemental Security Income disability, which is strictly means tested for disabled individuals.^23,24^ The ACA expanded access to Medicaid for SSDI recipients aged 19 to 64 years with low household incomes in addition to increasing Medicaid participation among those already eligible for coverage because of publicity, enrollment efforts, and other factors, which is sometimes referred to as the woodwork or welcome mat effect.^25,26^ Recent evidence suggests that Medicaid expansion was associated with increases in dual Medicare and Medicaid coverage for older adults with low household incomes and individuals with disabilities.^25^ This study supports these estimates, suggesting that patients with incident kidney failure had pronounced increases in dual Medicare and Medicaid coverage after expansion.

Second, the present study contributes new evidence to the implications of Medicaid expansion for changes in care use. Some policy makers hypothesized that expanded Medicaid coverage could reduce acute hospitalizations by bolstering access to primary care and other outpatient care. Most studies, however, have suggested that expanding Medicaid led to either increased hospitalizations or no changes in hospitalizations.^16,27,28,29^ Findings of the present study, which used national data and focused on a high-need and clinically complex patient population, suggest that Medicaid expansion was associated with significant reductions in all-cause and infection-related hospitalizations. These results align with past work finding that, among Medicare beneficiaries, particularly those with chronic conditions, lower cost sharing may increase access to effective outpatient care and generate offsetting reductions in acute hospitalizations.^30,31^ The present study’s findings suggest spillovers from the ACA’s Medicaid expansion to Medicare in the form of health benefits for Medicare enrollees and reduced spending on hospitalizations, the largest source of expenditures in the Medicare program.

Third, compared with other groups of Medicare beneficiaries, hospitalization rates are higher among people with kidney failure.^32^ There is evidence that the number of annual admissions and hospital days for all-cause, cardiovascular, and infection-related hospitalizations have been declining among all patients with kidney failure since 2007, which are trends observed in our study.^22^ For example, between 2007 and 2016, hospitalization rates for all patients undergoing dialysis decreased by approximately 15%, with 1 study noting that the decreases could be affected by changes in clinical care and policies that incentivize use of ambulatory care services.^1,33^ Findings of the present study suggest that, among patients aged 19 to 64 years initiating dialysis for incident kidney failure, reductions in hospitalizations and number of hospital days were more pronounced for patients residing in states that expanded Medicaid. The relative 8% decreases associated with Medicaid expansion were large in magnitude considering the secular trend between 2007 and 2016 was a 15% decline. This finding is critical because reducing hospitalizations among the population with kidney failure is a clinical and policy priority, including public reporting on hospitalization rates for dialysis facilities. Furthermore, Medicare is the primary payer for care among people with kidney failure, and hospitalizations account for approximately 40% of Medicare expenditures for patients undergoing dialysis.^22^ Significant decreases in 1-year mortality rates associated with Medicaid expansion support past findings^11^ and extend this work to Medicare beneficiaries initiating dialysis treatment.

Several studies have evaluated the role of health insurance coverage on access to care, and dual Medicare and Medicaid coverage may provide additional financial protection to access health services and medications for patients with low household incomes who are initiating dialysis.^8,34^ Although we did not identify significant changes in receipt of predialysis nephrology care by Medicaid expansion status, it is possible that increases in dual Medicare and Medicaid coverage changed accessibility to prescription drugs, primary care, or other specialty care that mitigated the number of hospitalizations and hospital days after dialysis initiation. The finding that Medicaid expansion was associated with increases in AVF may provide a mechanism for the outcomes studied: placement of permanent vascular access has been associated with fewer hospitalizations or emergency department use and lower mortality among patients with kidney failure.^35,36^ Initiating hemodialysis with an AVF has been associated with lower mortality compared with initiating dialysis with a catheter.^11,37^

### Limitations

This study has several limitations. First, the study sample was limited to individuals who initiated dialysis while covered by Medicare Part A insurance and may not be representative of the entire population with kidney failure. It is possible changes associated with Medicaid expansion would be different for those who initiate treatment without Medicare coverage. However, we found that nearly 40% of adults aged 19 to 64 years initiating maintenance dialysis had Medicare Part A coverage at the time of dialysis initiation. In addition, because the CMS 2728 is completed for patients initiating maintenance dialysis, individuals who received only dialysis for acute kidney failure and those who received dialysis in the hospital and died before initiating outpatient treatment were not included in the analysis. Second, we were restricted to events that occurred after a patient acquired Medicare coverage, and we did not have data on hospitalizations before enrollment in Medicare. Third, it is possible that we were unable to detect changes for subgroups because of smaller sample sizes. Fourth, the study period coincided with several changes in CMS rules around hospital stays (eg, Hospital Readmissions Reduction Program in 2012, introduction of the Two-Midnight Rule in 2013), but it is unlikely that these rules would differentially affect states by expansion status. Fifth, although the difference-in-differences approach and inclusion of state fixed effects account for secular trends and potential state-specific policy contexts, it is plausible that changes or differences in outcomes for patients who initiated treatment for kidney failure were not associated with a state’s decision to expand Medicaid. Sixth, for the outcome examining changes in dialysis type at initiation, it is possible that individuals switched their dialysis type (hemodialysis vs peritoneal) after initiation. Seventh, we were unable to assess differences in hospitalization-related out-of-pocket costs because the data set did not include information such as having supplemental coverage or hospital networks (for Medicare Advantage beneficiaries). However, additional work to understand the potential changes in out-of-pocket costs associated with Medicaid expansion among Medicare beneficiaries with kidney failure is warranted.

## Conclusions

In this cross-sectional study, Medicaid expansion was associated with decreases in Medicare-financed hospitalizations and fewer acute care hospital days in the first year after initiating dialysis, which is a high-risk period, as well as increases in dual Medicare and Medicaid coverage. This study suggests that there may have been favorable spillover outcomes of Medicaid expansion to Medicare-financed care, which is the primary payer for patients with kidney failure.

## Notes

Supplement. **eAppendix.** Detailed Descriptions of Main Model and Sensitivity Analyses **eFigure 1.** Changes in All-Cause Hospitalizations Within 6 Months of Dialysis Initiation **eFigure 2.** Changes in All-Cause Hospitalizations Within 12 Months of Dialysis Initiation **eFigure 3.** Changes in Receipt of Predialysis Nephrology Care and Home Dialysis **eFigure 4.** Changes in Receipt of Hemodialysis and Peritoneal Dialysis at Initiation **eFigure 5.** Changes in Cardiac-Related Hospitalizations and Hospital Days Within 3 Months of Dialysis Initiation **eFigure 6.** Changes in Cardiac-Related Hospitalizations and Hospital Days Within 6 Months of Dialysis Initiation **eFigure 7.** Changes in Cardiac-Related Hospitalizations and Hospital Days Within 12 Months of Dialysis Initiation **eFigure 8.** Changes in Infection-Related Hospitalizations and Hospital Days Within 3 Months of Dialysis Initiation **eFigure 9.** Changes in Infection-Related Hospitalizations and Hospital Days Within 6 Months of Dialysis Initiation **eFigure 10.** Changes in Infection-Related Hospitalizations and Hospital Days Within 12 Months of Dialysis Initiation **eFigure 11.** Falsification Test—All-Cause Hospital Admissions Within 3 Months of Dialysis Initiation **eFigure 12.** Falsification Test—All-Cause Hospital Admissions Within 6 Months of Dialysis Initiation **eFigure 13.** Falsification Test—All-Cause Hospital Admissions Within 12 Months of Dialysis Initiation **eFigure 14.** Falsification Test—All-Cause Hospital Days Within 3 Months of Dialysis Initiation **eFigure 15.** Falsification Test—All-Cause Hospital Days Within 6 Months of Dialysis Initiation **eFigure 16.** Falsification Test—All-Cause Hospital Days Within 12 Months of Dialysis Initiation **eTable 1.** Definitions of Expansion and Nonexpansion States **eTable 2.** Characteristics of Adult Persons Aged 19 to 64 With Kidney Failure, by Medicare Part A Coverage at Initiation, 2010 to 2018 **eTable 3.** Parallel Trends Assumption Test **eTable 4.** Changes in Composition of Patient Population by State Medicaid Expansion Status **eTable 5.** Sensitivity Analyses **eTable 6.** Postperiod Event Study Specification **eTable 7.** Differential Effects by Area-Level Poverty **eTable 8.** Differential Effects Between White and Black Patients **eTable 9.** Differential Effects Between White and Hispanic or Latino Patients **eTable 10.** Differential Effects Between White and Asian American Patients **eTable 11.** Differential Effects Between White and Other Race Patients **eTable 12.** Differential Effects Between Patients Aged 18 to 34 vs 35 to 44 Years **eTable 13.** Differential Effects Between Patients Aged 18 to 34 vs 45 to 54 Years **eTable 14.** Differential Effects Between Patients Aged 18 to 34 vs 55 to 64 Years **eTable 15.** Differential Effects by Sex **eTable 16.** Changes in Mortality Rates per 100 Patient-Years by State Expansion Status
eReferences.
