# Supplementary material for: Medicaid Expansion and Medicare-Financed Hospitalizations Among Adult Patients With Incident Kidney Failure
Source: JAMA Health Forum. 2022 Nov 4;3(11):e223878. doi: 10.1001/jamahealthforum.2022.3878 (PMC9636522; doi:10.1001/jamahealthforum.2022.3878)
Supplement: Supplement. — eAppendix. Detailed Descriptions of Main Model and Sensitivity Analyses eFigure 1. Changes in All-Cause Hospitalizations Within 6 Months of Dialysis Initiation eFigure 2. Changes in All-Cause Hospitalizations Within 12 Months of Dialysis Initiation eFigure 3. Changes in Receipt of Predialysis Nephrology Care and Home Dialysis eFigure 4. Changes in Receipt of Hemodialysis and Peritoneal Dialysis at Initiation eFigure 5. Changes in Cardiac-Related Hospitalizations and Hospital Days Within 3 Months of Dialysis Initiation eFigure 6. Changes in Cardiac-Related Hospitalizations and Hospital Days Within 6 Months of Dialysis Initiation eFigure 7. Changes in Cardiac-Related Hospitalizations and Hospital Days Within 12 Months of Dialysis Initiation eFigure 8. Changes in Infection-Related Hospitalizations and Hospital Days Within 3 Months of Dialysis Initiation eFigure 9. Changes in Infection-Related Hospitalizations and Hospital Days Within 6 Months of Dialysis Initiation eFigure 10. Changes in Infection-Related Hospitalizations and Hospital Days Within 12 Months of Dialysis Initiation eFigure 11. Falsification Test—All-Cause Hospital Admissions Within 3 Months of Dialysis Initiation eFigure 12. Falsification Test—All-Cause Hospital Admissions Within 6 Months of Dialysis Initiation eFigure 13. Falsification Test—All-Cause Hospital Admissions Within 12 Months of Dialysis Initiation eFigure 14. Falsification Test—All-Cause Hospital Days Within 3 Months of Dialysis Initiation eFigure 15. Falsification Test—All-Cause Hospital Days Within 6 Months of Dialysis Initiation eFigure 16. Falsification Test—All-Cause Hospital Days Within 12 Months of Dialysis Initiation eTable 1. Definitions of Expansion and Nonexpansion States eTable 2. Characteristics of Adult Persons Aged 19 to 64 With Kidney Failure, by Medicare Part A Coverage at Initiation, 2010 to 2018 eTable 3. Parallel Trends Assumption Test eTable 4. Changes in Composition of Patient Population by State Medicaid Expansion Statu [file jamahealthforum-e223878-s001.pdf]

Supplementary Online Content

Nguyen KH, Lee Y, Thorsness R, et al. Medicaid expansion and Medicare-financed hospitalizations among adult patients with incident kidney failure. *JAMA Health Forum.* 3(11):e223878. doi:10.1001/jamahealthforum.2022.3878

eAppendix. Detailed Descriptions of Main Model and Sensitivity Analyses
eFigure 1. Changes in All-Cause Hospitalizations Within 6 Months of Dialysis Initiation
eFigure 2. Changes in All-Cause Hospitalizations Within 12 Months of Dialysis Initiation
eFigure 3. Changes in Receipt of Predialysis Nephrology Care and Home Dialysis
eFigure 4. Changes in Receipt of Hemodialysis and Peritoneal Dialysis at Initiation
eFigure 5. Changes in Cardiac-Related Hospitalizations and Hospital Days Within 3 Months of Dialysis Initiation
eFigure 6. Changes in Cardiac-Related Hospitalizations and Hospital Days Within 6 Months of Dialysis Initiation
eFigure 7. Changes in Cardiac-Related Hospitalizations and Hospital Days Within 12 Months of Dialysis Initiation
eFigure 8. Changes in Infection-Related Hospitalizations and Hospital Days Within 3 Months of Dialysis Initiation
eFigure 9. Changes in Infection-Related Hospitalizations and Hospital Days Within 6 Months of Dialysis Initiation
eFigure 10. Changes in Infection-Related Hospitalizations and Hospital Days Within 12 Months of Dialysis Initiation
eFigure 11. Falsification Test—All-Cause Hospital Admissions Within 3 Months of Dialysis Initiation
eFigure 12. Falsification Test—All-Cause Hospital Admissions Within 6 Months of Dialysis Initiation
eFigure 13. Falsification Test—All-Cause Hospital Admissions Within 12 Months of Dialysis Initiation
eFigure 14. Falsification Test—All-Cause Hospital Days Within 3 Months of Dialysis Initiation
eFigure 15. Falsification Test—All-Cause Hospital Days Within 6 Months of Dialysis Initiation
eFigure 16. Falsification Test—All-Cause Hospital Days Within 12 Months of Dialysis Initiation
eTable 1. Definitions of Expansion and Nonexpansion States
eTable 2. Characteristics of Adult Persons Aged 19 to 64 With Kidney Failure, by Medicare Part A Coverage at Initiation, 2010 to 2018
eTable 3. Parallel Trends Assumption Test
eTable 4. Changes in Composition of Patient Population by State Medicaid Expansion Status
eTable 5. Sensitivity Analyses
eTable 6. Postperiod Event Study Specification
eTable 7. Differential Effects by Area-Level Poverty

© 2022 Nguyen KH et al. *JAMA Health Forum.*

eTable 8. Differential Effects Between White and Black Patients
eTable 9. Differential Effects Between White and Hispanic or Latino Patients
eTable 10. Differential Effects Between White and Asian American Patients
eTable 11. Differential Effects Between White and Other Race Patients
eTable 12. Differential Effects Between Patients Aged 18 to 34 vs 35 to 44 Years
eTable 13. Differential Effects Between Patients Aged 18 to 34 vs 45 to 54 Years
eTable 14. Differential Effects Between Patients Aged 18 to 34 vs 55 to 64 Years
eTable 15. Differential Effects by Sex
eTable 16. Changes in Mortality Rates per 100 Patient-Years by State Expansion Status
eReferences.

This supplementary material has been provided by the authors to give readers additional information about their work.

© 2022 Nguyen KH et al. *JAMA Health Forum.*

eAppendix. Detailed Descriptions of Main Model and Sensitivity Analyses

Main Model. Our main model was a linear regression model adjusted for age, sex, race/ethnicity, primary cause of kidney failure, presence of congestive heart failure, atherosclerotic heart disease, other cardiac disease, hypertension, diabetes, diabetic retinopathy, cancer, smoking status, or alcohol dependence at dialysis initiation. Model also adjusts for hemoglobin, albumin, and body mass index at dialysis initiation. For observations with missing covariates, we used the mean value of the covariates for non-missing observations. Included state and year-quarter fixed effects. Post-period is defined based on each state's implementation date, which for most states was January 1, 2014. However, some states expanded after this date (see below and eTable 1). A state's post-expansion period ranges between 2 years (Louisiana) include up to 5 years (for states that expanded January 1, 2014). Our main model excluded five early expanding states (District of Columbia, Delaware, Massachusetts, New York, and Vermont). Below we present the model in equation form:

$$(1) \quad y_{ijqt} = \beta_0 + \beta_1 Expansion_j + \beta_2 Post_{jqt} + \beta_3 \left(Expansion_j * Post_{jqt}\right) + X_i + \delta_j + \nu_{qt} + \varepsilon_{ijqt}$$

for each outcome y for beneficiary i living in state j at quarter q of year t. In this equation, $Expansion_j$ is an indicator for whether the state expanded Medicaid, $Post_{jqt}$ is an indicator for post-expansion (vs. pre-expansion), X_i as a vector of beneficiary-level sociodemographic and clinical variables, δ_j is a state fixed effect, and ν_{qt} is a year-quarter fixed effect. The coefficient of interest for measuring differential changes over time by state Medicaid expansion status is β_3.

Poisson Model: Adjusts using the same covariates as **Main Model** but applies Poisson model, rather than linear (only for number of hospital days outcomes)

No Lab Values: Adjusts for same covariates as **Main Model**, but does not adjust for hemoglobin and albumin values.

Exclude Missing Lab Values: Adjusts for same covariates as **Main Model** but excludes patients missing values for hemoglobin or albumin.

Missing Indicators for Labs: Adjusts for same covariates as **Main Model** but created quintiles for hemoglobin and albumin values, and included an indicator when data was missing.

Includes Original Reason for Medicare Entitlement: Adjusts for same covariates as **Main Model** but also includes original reason for Medicare entitlement.

Exclude Late Expanders: Excludes observations for patients living in Alaska, Indiana, Louisiana, Michigan, Montana, New Hampshire, and Pennsylvania (n=25,939, or 13.6% of sample)

Include Early Expanders: Added observations for patients living in District of Columbia, Delaware, Massachusetts, New York, or Vermont (n=14,688)

© 2022 Nguyen KH et al. *JAMA Health Forum.*

eFigure 1. Changes in All-Cause Hospitalizations Within 6 Months of Dialysis Initiation

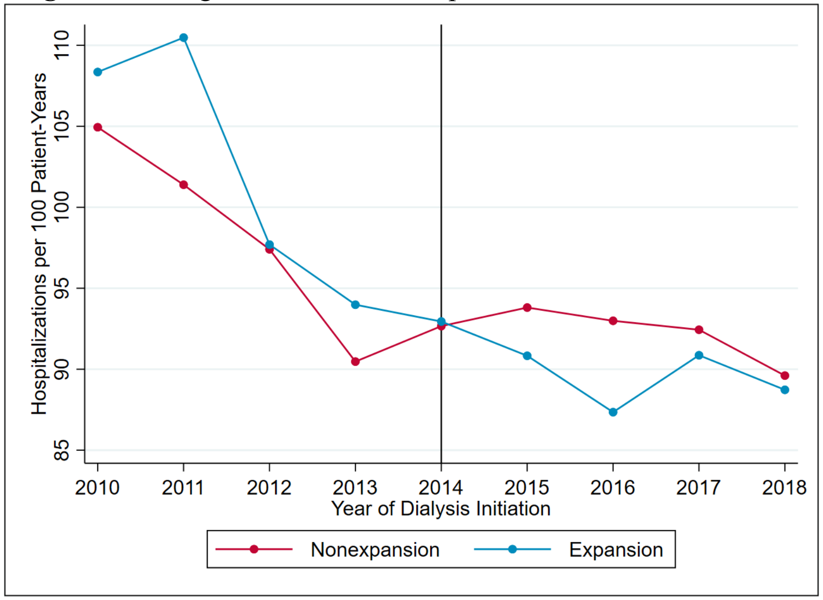

Notes. Expansion states limited to those that expanded in 2014 and excludes those that did not expand in 2015 onward. Vertical line represents Medicaid expansion in 2014.

© 2022 Nguyen KH et al. *JAMA Health Forum.*

eFigure 2. Changes in All-Cause Hospitalizations Within 12 Months of Dialysis Initiation

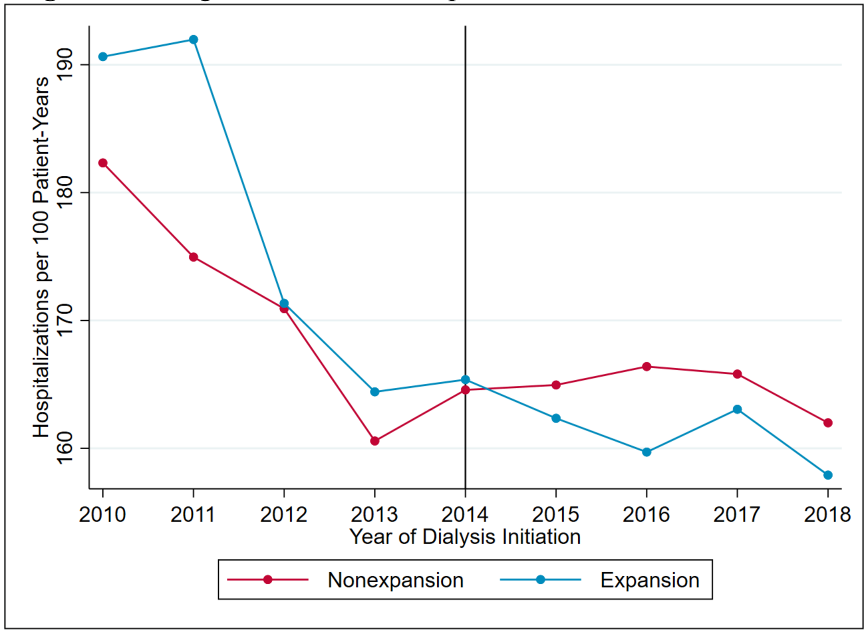

Notes. Expansion states limited to those that expanded in 2014 and excludes those that did not expand in 2015 onward. Vertical line represents Medicaid expansion in 2014.

© 2022 Nguyen KH et al. *JAMA Health Forum.*

eFigure 3. Changes in Receipt of Predialysis Nephrology Care and Home Dialysis

Panel A: Receipt of Pre-Dialysis Nephrology Care

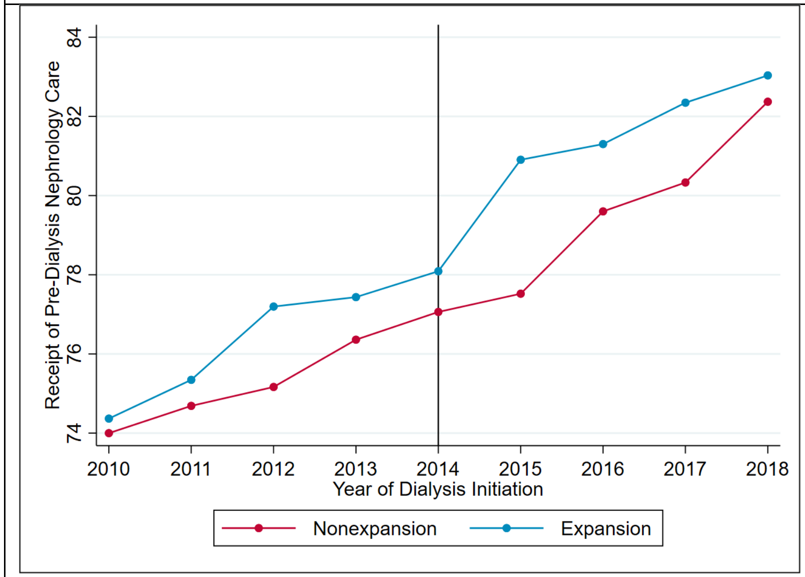

Panel B: Receipt of Home Dialysis

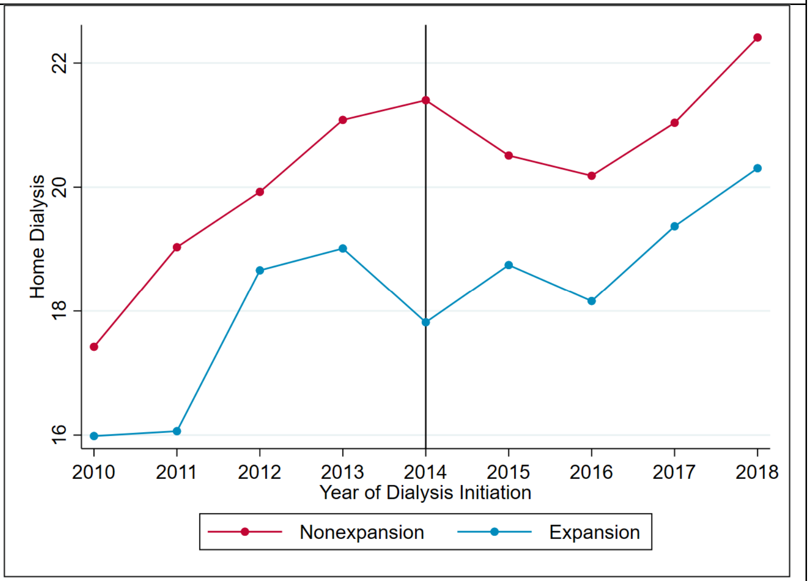

Expansion states limited to those that expanded in 2014 and excludes those that did not expand in 2015 onward. Vertical line represents Medicaid expansion in 2014.

© 2022 Nguyen KH et al. *JAMA Health Forum.*

eFigure 4. Changes in Receipt of Hemodialysis and Peritoneal Dialysis at Initiation

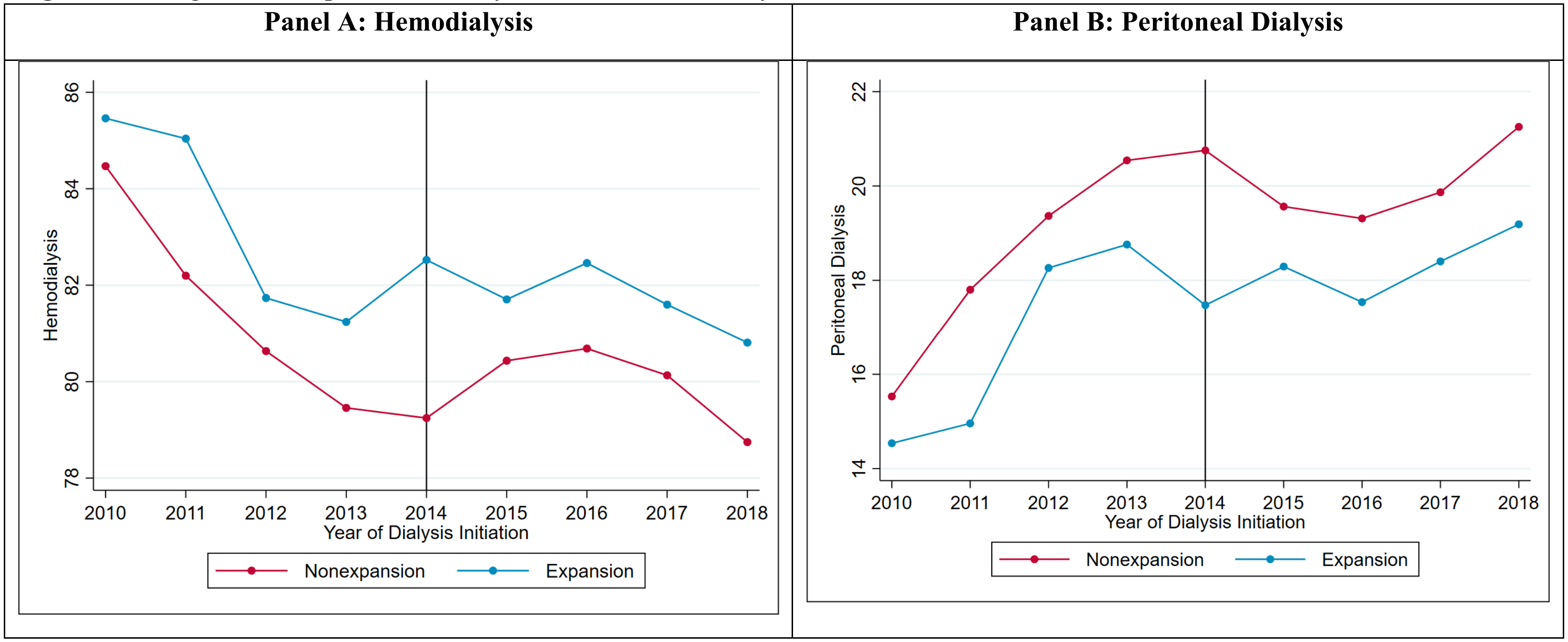

Expansion states limited to those that expanded in 2014 and excludes those that did not expand in 2015 onward. Vertical line represents Medicaid expansion in 2014.

© 2022 Nguyen KH et al. *JAMA Health Forum.*

eFigure 5. Changes in Cardiac-Related Hospitalizations and Hospital Days Within 3 Months of Dialysis Initiation

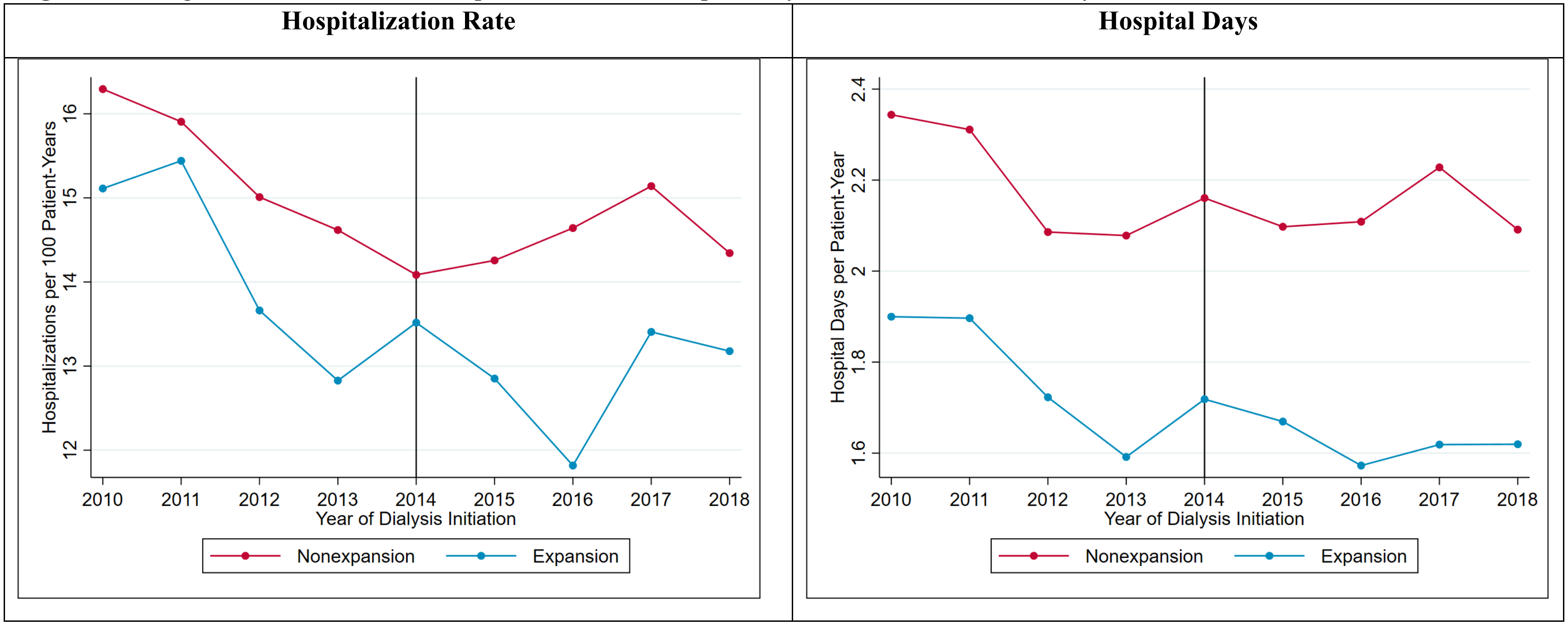

Notes. Expansion states limited to those that expanded in 2014 and excludes those that did not expand in 2015 onward. Vertical line represents Medicaid expansion in 2014.

© 2022 Nguyen KH et al. *JAMA Health Forum.*

eFigure 6. Changes in Cardiac-Related Hospitalizations and Hospital Days Within 6 Months of Dialysis Initiation

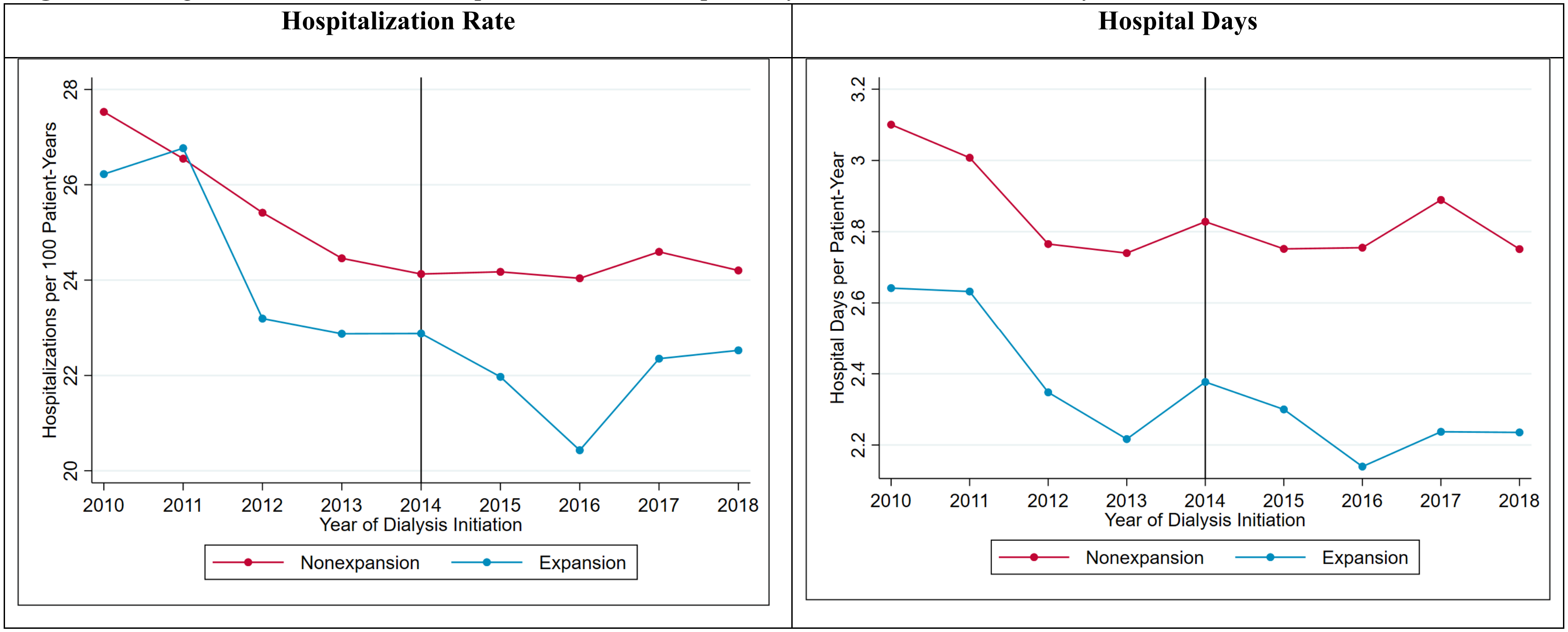

Notes. Expansion states limited to those that expanded in 2014 and excludes those that did not expand in 2015 onward. Vertical line represents Medicaid expansion in 2014.

© 2022 Nguyen KH et al. *JAMA Health Forum.*

eFigure 7. Changes in Cardiac-Related Hospitalizations and Hospital Days Within 12 Months of Dialysis Initiation

Hospitalization Rate	Hospital Days
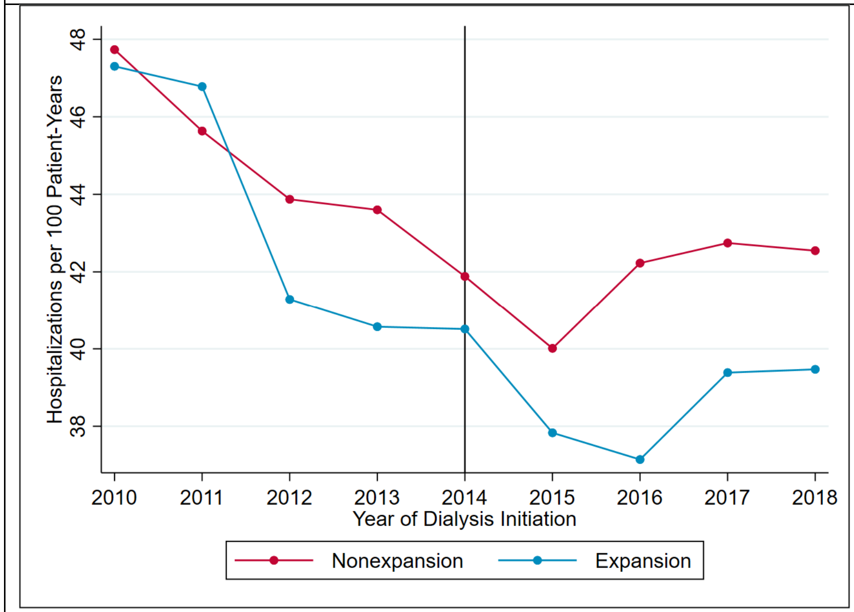	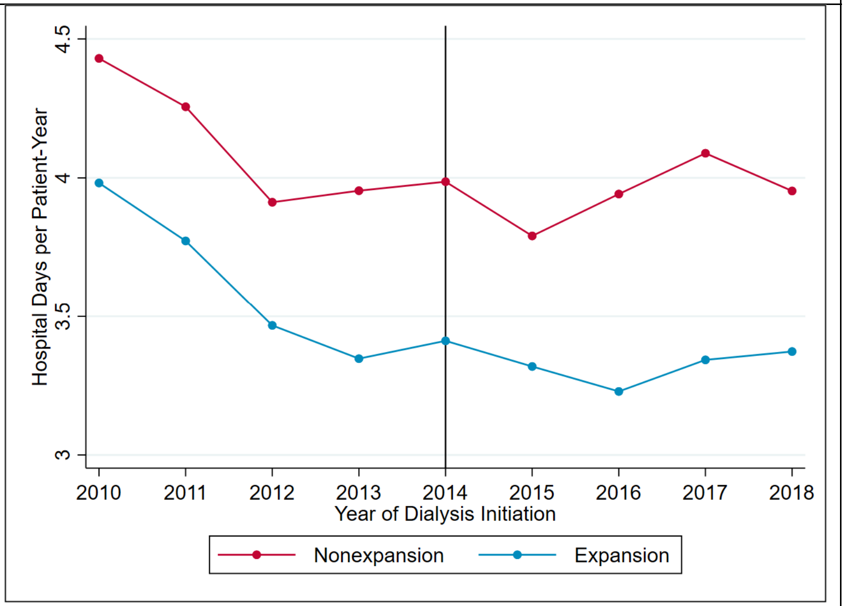

Notes. Expansion states limited to those that expanded in 2014 and excludes those that did not expand in 2015 onward. Vertical line represents Medicaid expansion in 2014.

© 2022 Nguyen KH et al. *JAMA Health Forum.*

eFigure 8. Changes in Infection-Related Hospitalizations and Hospital Days Within 3 Months of Dialysis Initiation

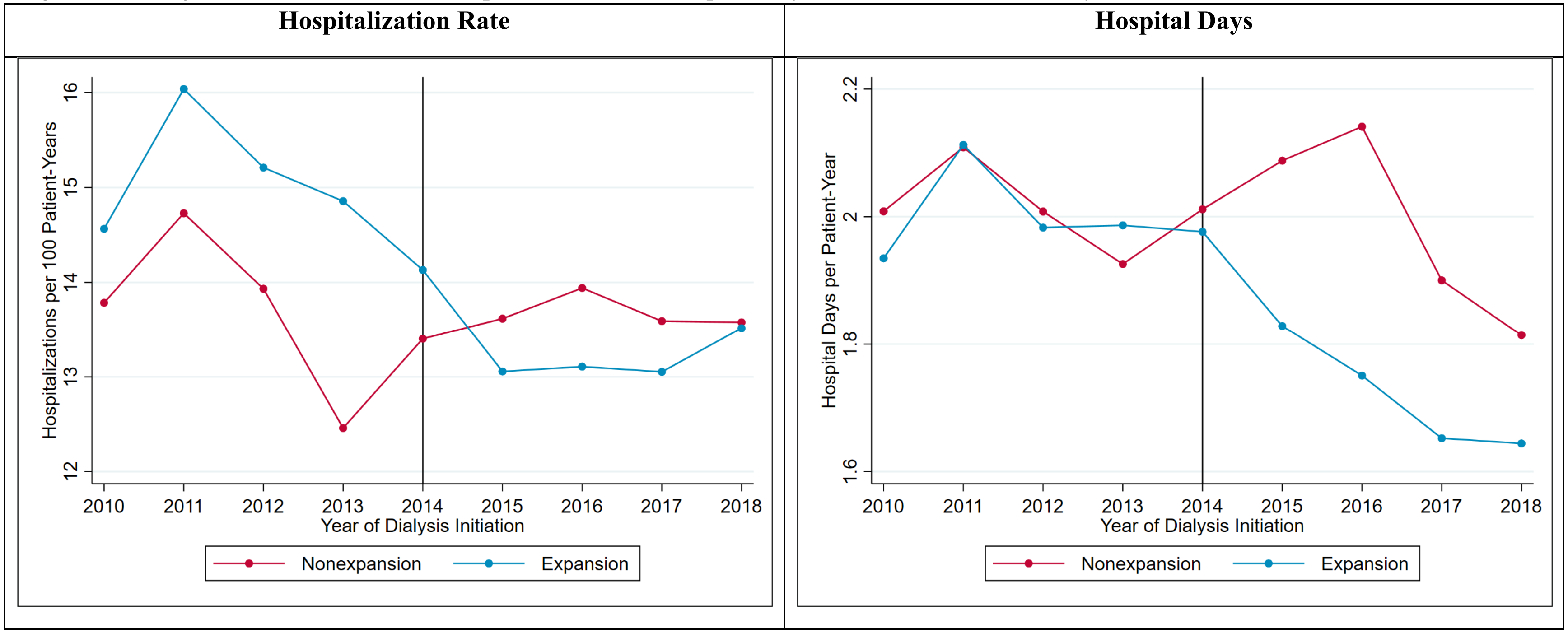

Notes. Expansion states limited to those that expanded in 2014 and excludes those that did not expand in 2015 onward. Vertical line represents Medicaid expansion in 2014.

© 2022 Nguyen KH et al. *JAMA Health Forum.*

eFigure 9. Changes in Infection-Related Hospitalizations and Hospital Days Within 6 Months of Dialysis Initiation

Hospitalization Rate	Hospital Days

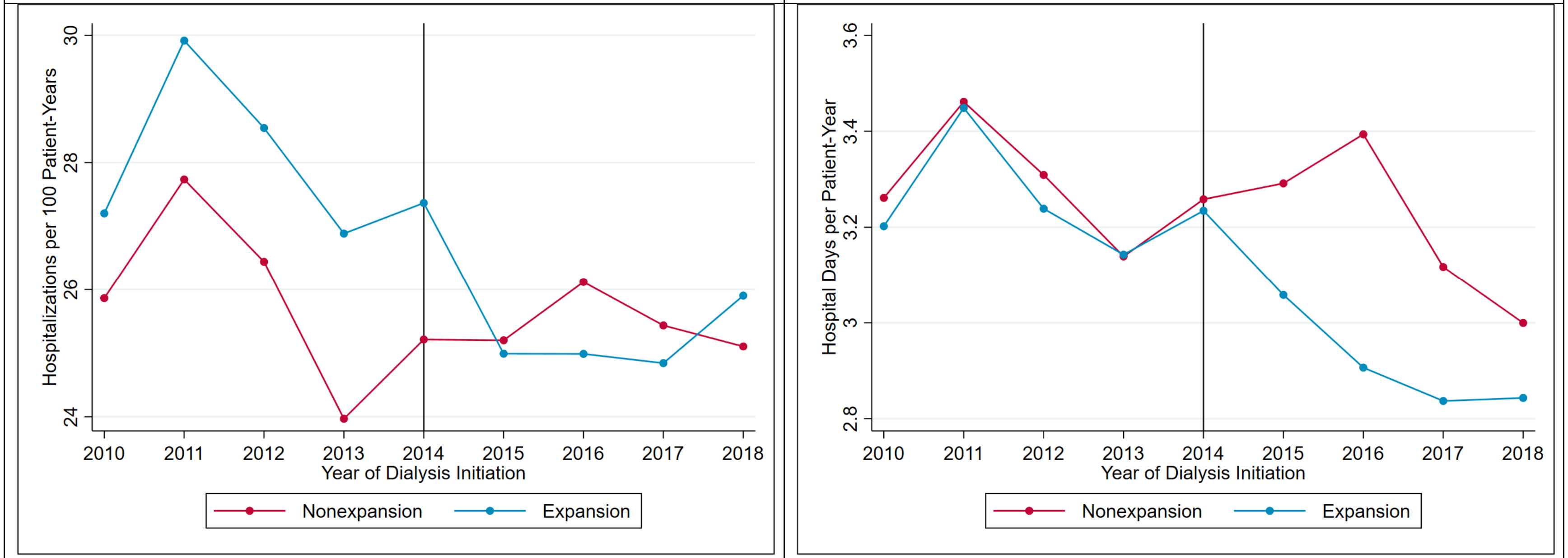

Notes. Expansion states limited to those that expanded in 2014 and excludes those that did not expand in 2015 onward. Vertical line represents Medicaid expansion in 2014.

© 2022 Nguyen KH et al. *JAMA Health Forum.*

eFigure 10. Changes in Infection-Related Hospitalizations and Hospital Days Within 12 Months of Dialysis Initiation

**Hospitalization Rate**	**Hospital Days**

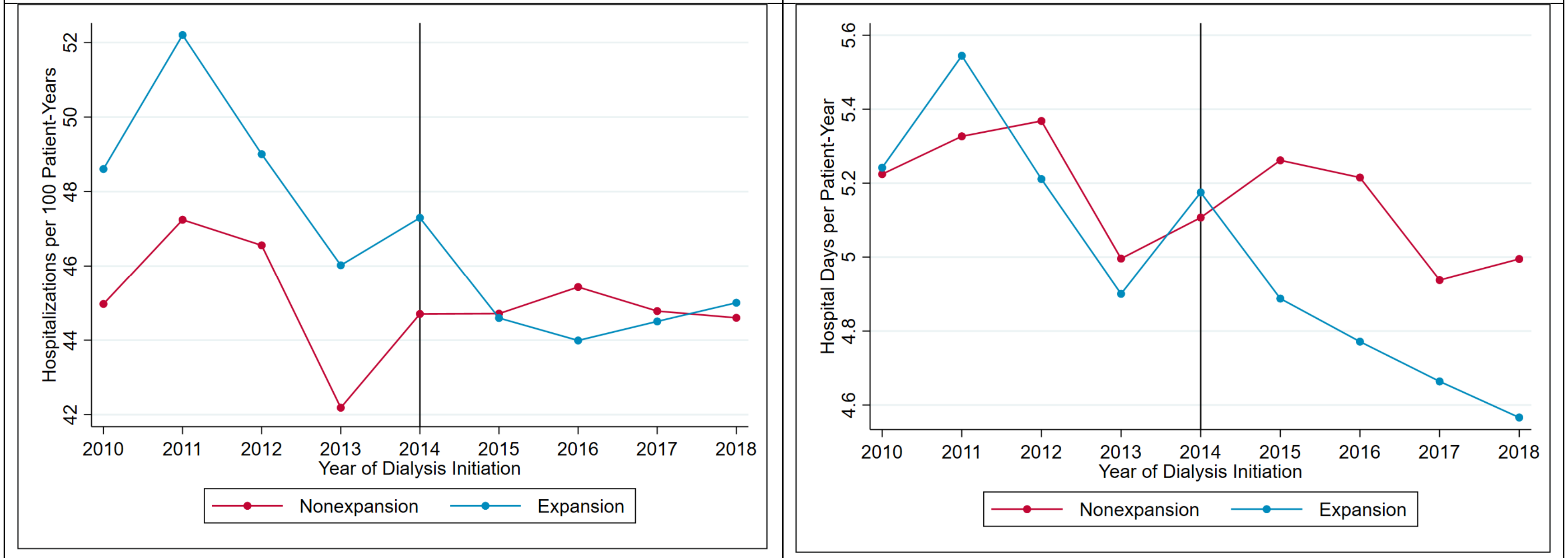

Notes. Expansion states limited to those that expanded in 2014 and excludes those that did not expand in 2015 onward. Vertical line represents Medicaid expansion in 2014.

© 2022 Nguyen KH et al. *JAMA Health Forum.*

eFigure 11. Falsification Test—All-Cause Hospital Admissions Within 3 Months of Dialysis Initiation

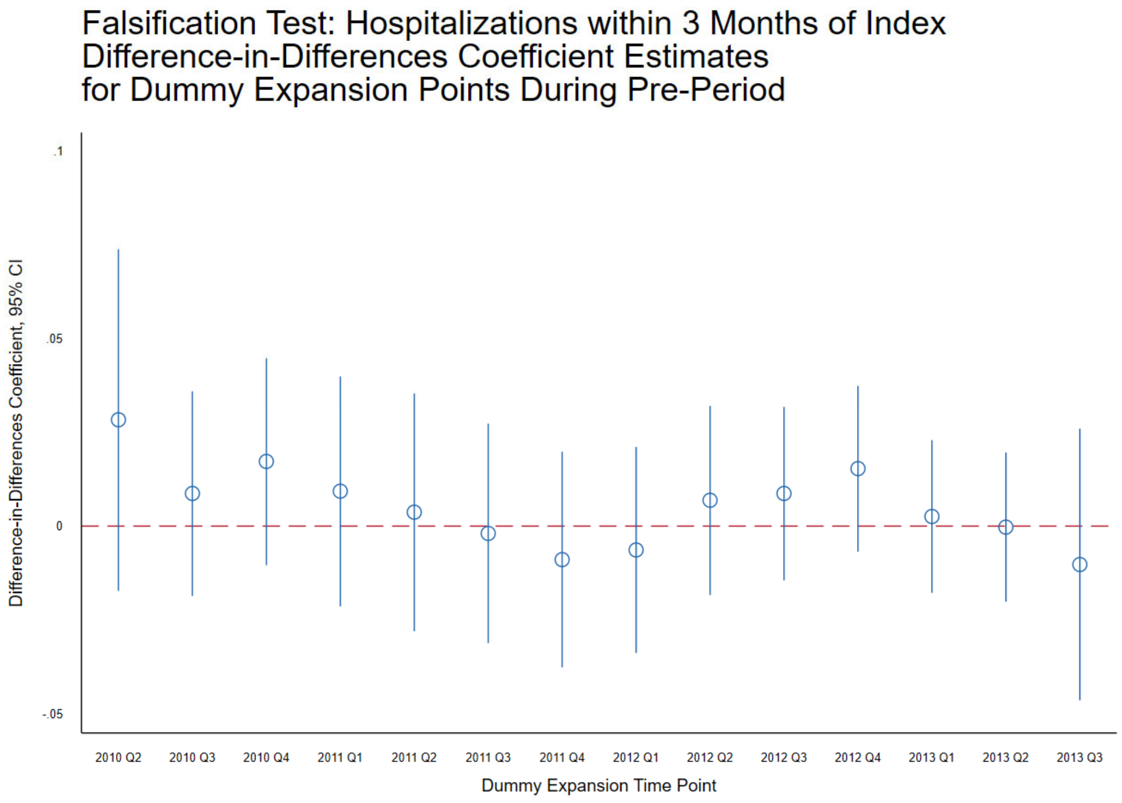

© 2022 Nguyen KH et al. *JAMA Health Forum.*

eFigure 12. Falsification Test—All-Cause Hospital Admissions Within 6 Months of Dialysis Initiation

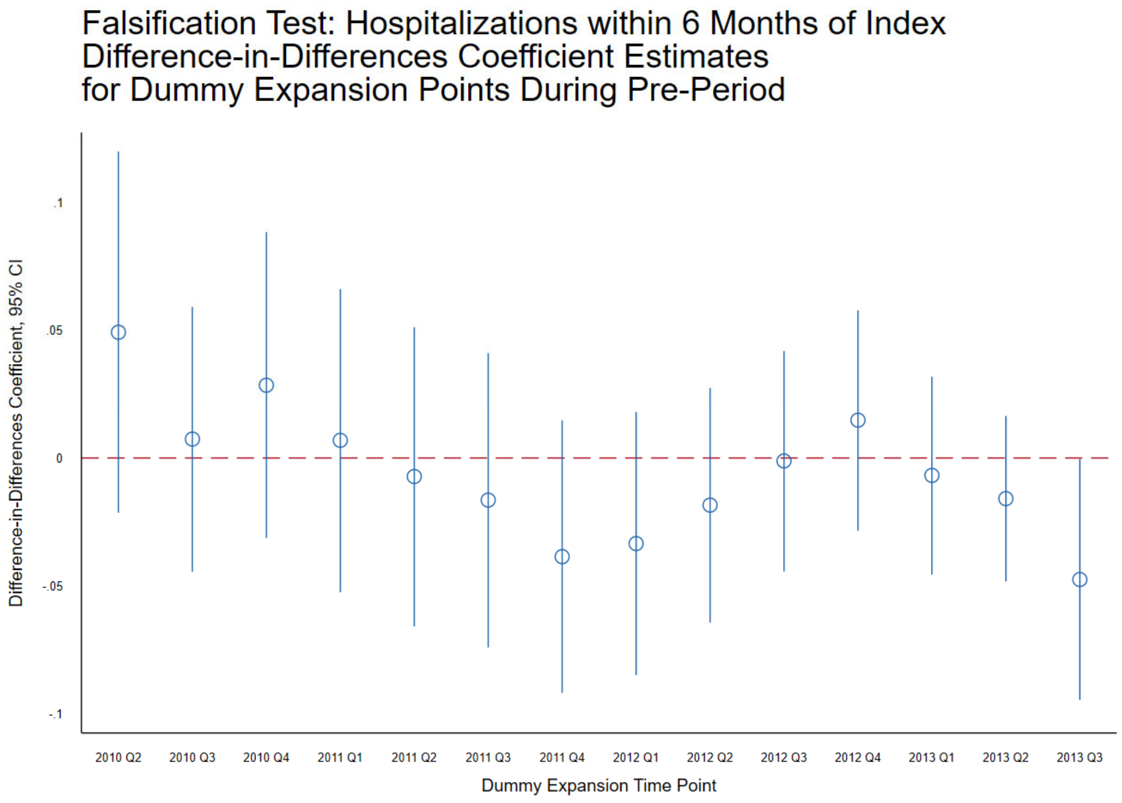

© 2022 Nguyen KH et al. *JAMA Health Forum.*

eFigure 13. Falsification Test—All-Cause Hospital Admissions Within 12 Months of Dialysis Initiation

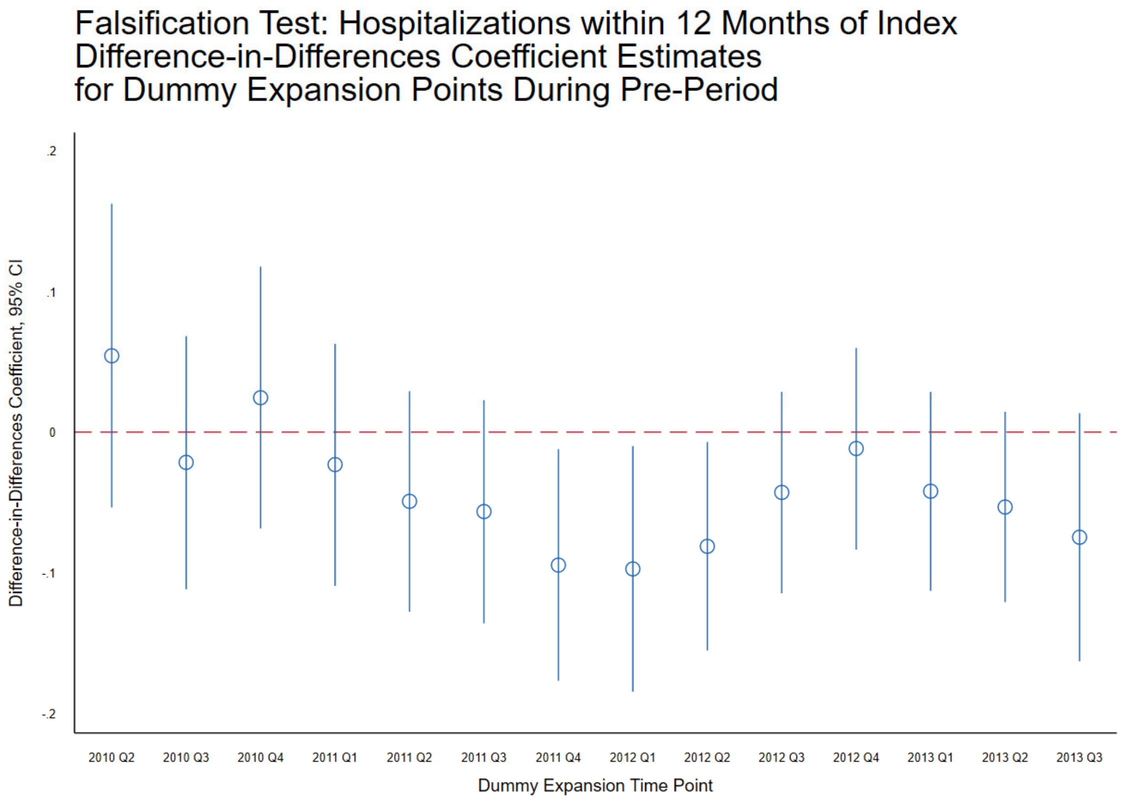

© 2022 Nguyen KH et al. *JAMA Health Forum.*

eFigure 14. Falsification Test—All-Cause Hospital Days Within 3 Months of Dialysis Initiation

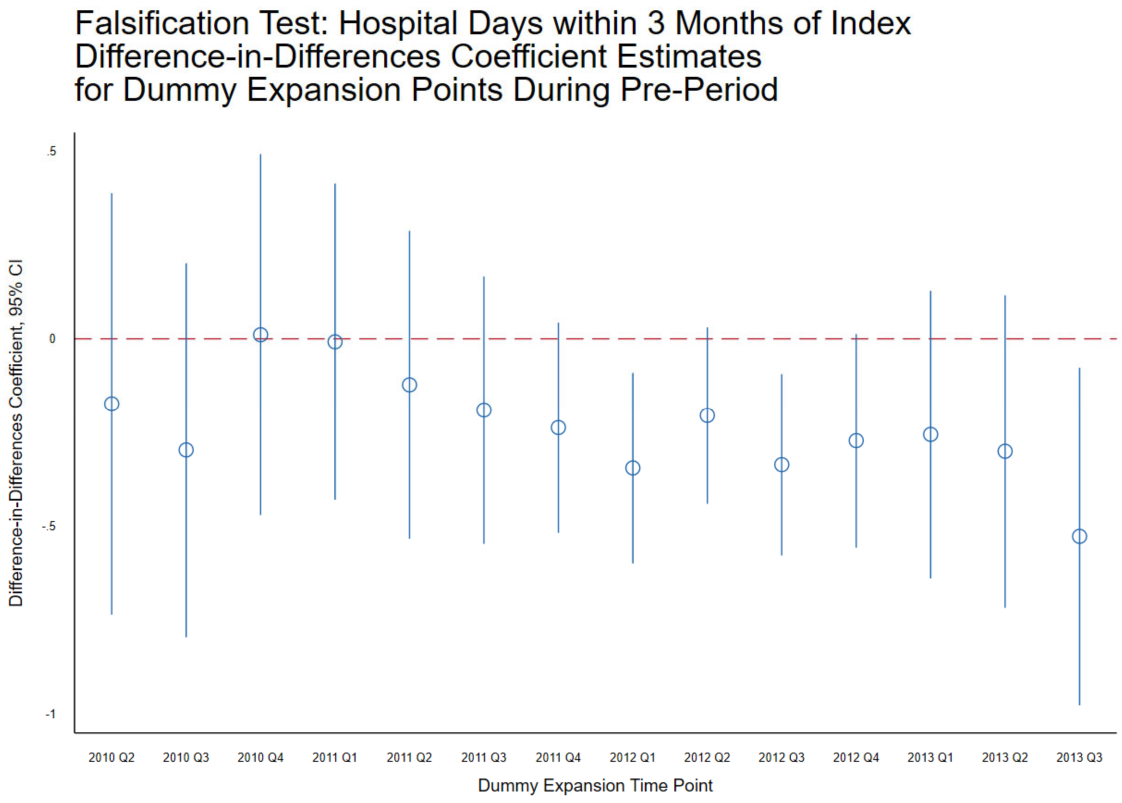

© 2022 Nguyen KH et al. *JAMA Health Forum.*

eFigure 15. Falsification Test—All-Cause Hospital Days Within 6 Months of Dialysis Initiation

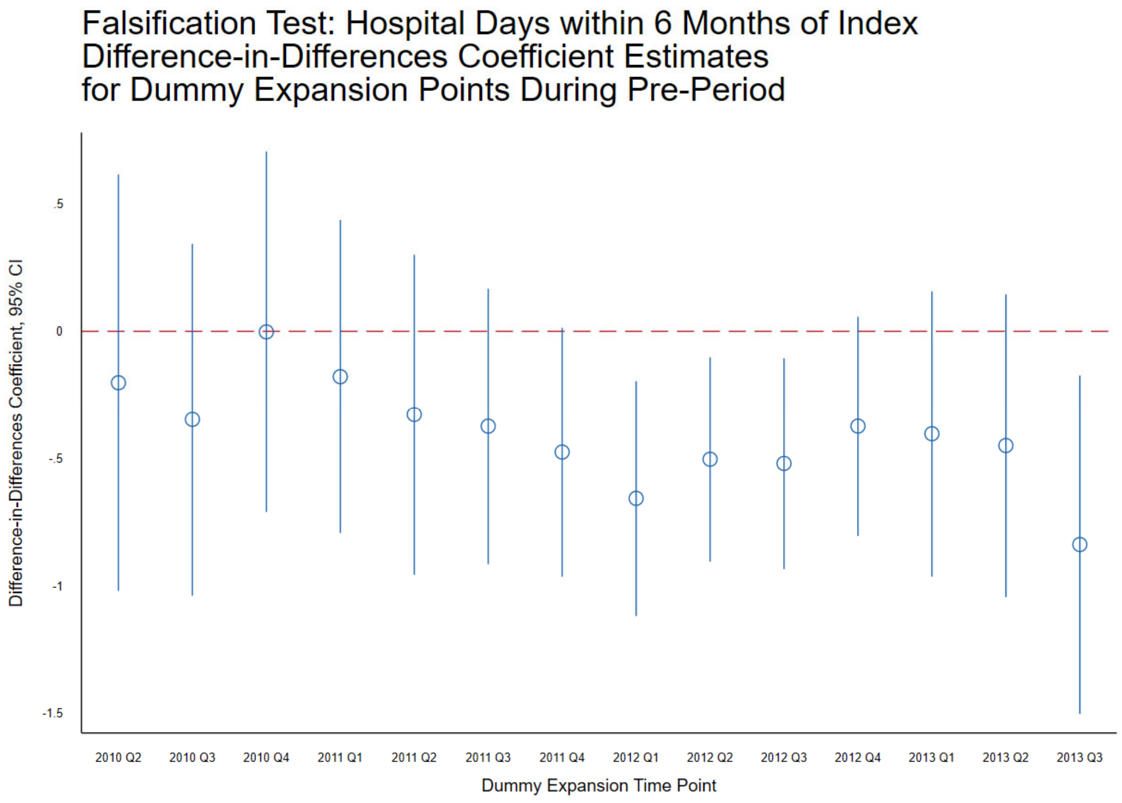

© 2022 Nguyen KH et al. *JAMA Health Forum.*

eFigure 16. Falsification Test—All-Cause Hospital Days Within 12 Months of Dialysis Initiation

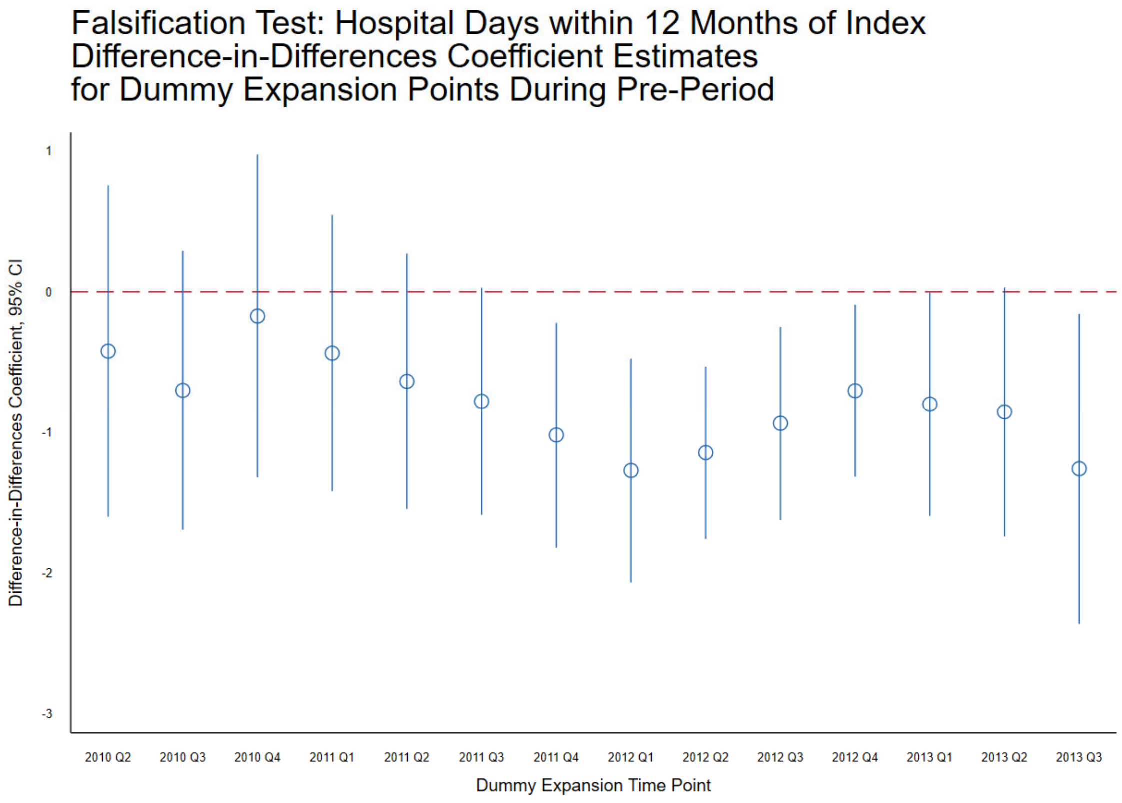

© 2022 Nguyen KH et al. *JAMA Health Forum.*

eTable 1. Definitions of Expansion and Nonexpansion States

Patients were defined as living in Medicaid expansion state if they initiated dialysis in a state that expanded Medicaid between January 1, 2014 and December 31, 2018. Consistent with Wherry and Miller 2017, we excluded individuals living in states that extended Medicaid eligibility (or similar coverage) to adults with incomes of up to 100% of the federal poverty line or greater in the pre-expansion period (2010-2013). These states were the District of Columbia, Delaware, Massachusetts, New York, and Vermont. Below we present the dates of Medicaid expansion for each state.

State(s)	Date of ACA Medicaid Expansion[1]	Year Considered Expanded
Arizona, Arkansas, California, Colorado, Connecticut, Delaware,* D.C.,* Hawaii, Illinois, Iowa, Kentucky, Maryland, Massachusetts,* Minnesota, Nevada, New Jersey, New Mexico, New York,* North Dakota, Ohio, Oregon, Rhode Island, Vermont,* Washington, West Virginia	January 1, 2014	2014
Michigan	April 1, 2014	2014
New Hampshire	August 15, 2014	2015
Pennsylvania	January 1, 2015	2015
Indiana	February 1, 2015	2015
Alaska	September 1, 2015	2016
Montana	January 1, 2016	2016
Louisiana	July 1, 2016	2017

Notes. *Indicates early expanding state, per Wherry and Miller 2017.[2] States that did not expand Medicaid during the study period: Alabama, Florida, Georgia, Idaho, Kansas, Maine, Mississippi, Missouri, Nebraska, North Carolina, Oklahoma, South Carolina, South Dakota, Tennessee, Texas, Utah, Virginia, Wisconsin, Wyoming.

© 2022 Nguyen KH et al. *JAMA Health Forum.*

eTable 2. Characteristics of Adult Persons Aged 19 to 64 With Kidney Failure, by Medicare Part A Coverage at Initiation, 2010 to 2018

	**No Part A Coverage**	**Part A Coverage**
**Number of patients, n**	**N=349,950**	**N=212,221**
**Age, mean (SD)**	50.08 (10.80)	54.04 (9.91)
**Age category, %**		
19-34	38,487 (11.0%)	11,830 (5.6%)
35-44	56,332 (16.1%)	23,958 (11.3%)
45-54	106,135 (30.3%)	57,980 (27.3%)
55-64	148,996 (42.6%)	118,453 (55.8%)
**Sex, %**		
Male	210,044 (60.0%)	125,157 (59.0%)
Female	139,906 (40.0%)	87,064 (41.0%)
**Race/ethnicity, %**		
African American/Black non-Hispanic	112,426 (32.1%)	69,399 (32.7%)
Asian non-Hispanic	16,379 (4.7%)	5,035 (2.4%)
Hispanic/Latino	75,595 (21.6%)	27,541 (13.0%)
White non-Hispanic	134,438 (38.4%)	106,488 (50.2%)
Other Race	11,112 (3.2%)	3,758 (1.8%)
**Primary cause of kidney failure, %**		
Diabetes	161,980 (46.3%)	100,214 (47.2%)
Hypertension	90,472 (25.9%)	43,795 (20.6%)
Other	97,498 (27.9%)	68,190 (32.1%)
**Comorbid conditions, %**		
Congestive heart failure	67,177 (19.2%)	55,355 (26.1%)
Atherosclerotic heart disease	28,076 (8.0%)	27,114 (12.8%)
Other cardiac disease	37,688 (10.8%)	35,084 (16.5%)
Hypertension	291,325 (83.2%)	186,425 (87.8%)
Diabetes	180,877 (51.7%)	131,581 (62.0%)
Diabetic retinopathy	29,424 (8.4%)	20,935 (9.9%)
Cancer	11,669 (3.3%)	9,819 (4.6%)
Obesity (BMI > 30 kg/m^2)	69,717 (19.9%)	101,414 (47.8%)
**Current smoker, %**	27,481 (7.9%)	18,380 (8.7%)
**Alcohol dependence, %**	8,068 (2.3%)	3,593 (1.7%)
**Hemoglobin, g/dl mean (SD)**	9.48 (10.25)	9.64 (11.07)
**Serum albumin, g/dl mean (SD)**	3.18 (2.82)	3.21 (2.80)

Notes. p<0.001 for all comparisons using Pearson's chi-square tests (categorical variables) or t-tests (continuous variables). Analysis includes persons residing in early expanding states.

© 2022 Nguyen KH et al. *JAMA Health Forum.*

eTable 3. Parallel Trends Assumption Test

	Estimate	95% Confidence Interval
**Dual Medicare and Medicaid coverage**	0.17	-0.0, 0.35
**Receipt of pre-dialysis nephrology care**	0.17	-0.01, 0.35
**Arteriovenous fistula or graft**	0.02	-0.10, 0.15
**Home Dialysis**	-0.02	-0.18, 0.13
**Dialysis Type at Initiation**		
Peritoneal Dialysis	-0.04	-0.19, 0.12
Hemodialysis	0.04	-0.12, 0.19
**Hospital admissions per 100 patient-years**		
3 months of dialysis initiation	0.04	-0.19, 0.27
6 months of dialysis initiation	-0.13	-0.62, 0.35
12 months of dialysis initiation	-0.62	-1.42, 0.17
**Number of hospital days per 100 patient-years**		
3 months of dialysis initiation	-0.03	-0.06, 0.001
6 months of dialysis initiation	-0.05*	-0.10, -0.005
12 months of dialysis initiation	-0.10*	-0.18, -0.02
**Cardiac Hospitalizations**		
**Hospital Admissions per 100 Patient-Years**		
3 months of dialysis initiation	-0.02	-0.14, 0.09
6 months of dialysis initiation	-0.05	-0.22, 0.13
12 months of dialysis initiation	-0.27	-0.57, 0.04
**Number of hospital days**		
3 months of dialysis initiation	-0.01	-0.02, 0.01
6 months of dialysis initiation	-0.01	-0.03, 0.01
12 months of dialysis initiation	-0.02	-0.05, 0.01
**Infection-Related Hospitalizations**		
**Hospital Admissions per 100 Patient-Years**		
3 months of dialysis initiation	0.09	-0.03, 0.21
6 months of dialysis initiation	0.09	-0.12, 0.30
12 months of dialysis initiation	-0.05	-0.35, 0.24
**Number of hospital days**		
3 months of dialysis initiation	0.46	-0.01, 0.02
6 months of dialysis initiation	0.0	-0.03, 0.03
12 months of dialysis initiation	-0.02	-0.06, 0.01

*p<0.05. p=0.031 for “Number of all-cause hospital days per 100-patient years within 6 months of treatment initiation” and p=0.0011 for “Number of all-cause hospital days per 100-patient years within 12 months of treatment initiation.” All estimates are the regression coefficients of an interaction term between an indicator variable for whether the patient resided in a Medicaid expansion state and a linear time trend for pre-2014 quarters (Expansion x Time).

© 2022 Nguyen KH et al. *JAMA Health Forum.*

eTable 4. Changes in Composition of Patient Population by State Medicaid Expansion Status

Characteristic	Coefficient (95% CI)
**Age**	0.0 (-0.02, 0.02)
**Female**	-0.80 (-1.70, 0.01)
**Race/Ethnicity**	
White	-1.58 (-2.45, -0.71)***
Black	0.76 (-0.04, 1.57)
Hispanic/Latino	0.53 (-0.03, 1.09)
Asian	0.10 (-0.16, 0.36)
Other	0.19 (-0.04, 0.43)
**Primary cause of kidney failure**	
Diabetes	-0.13 (-1.04, 0.78)
Hypertension	-0.66 (-1.40, 0.08)
Other	0.84 (0.03, 1.65)*
**Comorbid conditions**	
Congestive heart failure	0.15 (-0.65, 0.95)
Atherosclerotic heart disease	-0.36 (-0.97, 0.23)
Other cardiac disease	1.02 (0.35, 1.69)**
Hypertension	-0.56 (-0.04, 1.16)
Diabetes	-0.71 (-1.60, 0.18)
Diabetic retinopathy	0.67 (0.22, 1.33)*
Cancer	0.30 (-0.07, 0.68)
Obese	-1.05 (-1.97, -0.13)*
**Current smoker**	0.28 (-0.24, 0.81)
**Alcohol dependence**	0.14 (-0.10, 0.39)
**Hemoglobin**	0.26 (0.00, 0.51)*
**Serum albumin**	0.0 (-0.07, 0.07)
**Area-level poverty**	0.20 (0.0, 0.39)*

Notes. *p<0.05, **p<0.01, ***p<0.001. Coefficient estimates reflect compositional shifts over time by state Medicaid expansion status. Model includes indicators for Medicaid expansion, post-expansion period, and their interaction (Medicaid x post) and includes state and time (year-quarter) fixed effects. Changes are measured in percentage point terms for all characteristics except mean age, hemoglobin and albumin.

© 2022 Nguyen KH et al. *JAMA Health Forum.*

eTable 5. Sensitivity Analyses

	Unadjusted	Main Model	Poisson Model	No Lab Values	Exclude Missing Lab Values	Missing Indicator for Labs	Includes Original Reason for Medicare Entitlement	Exclude Late Expanders	Include Early Expanders
**Primary Outcomes**									
**All-Cause Hospitalizations**									
**Hospital admissions per 100 patient-years**									
3 months of dialysis initiation	-4.05** (-6.41 - -1.70)	-4.24** (-6.70 - -1.78)		-4.17** (-6.61 - -1.72)	-3.55* (-6.75 - -0.35)	-4.63*** (-7.01 - -2.25)	-4.30** (-6.83 - -1.77)	-4.70** (-7.52 - -1.89)	-3.59** (-6.16 - -1.03)
6 months of dialysis initiation	-5.57* (-9.93 - -1.21)	-5.79* (-10.36 - -1.23)		-5.64* (-10.19 - -1.09)	-4.17 (-9.75 - 1.41)	-6.52** (-10.89 - -2.15)	-5.96* (-10.59 - -1.34)	-5.97* (-11.04 - -0.91)	-4.63 (-9.51 - 0.246)
12 months of dialysis initiation	-8.93* (-17.03 - -0.83)	-9.27* (-17.81 - -0.73)		-8.95* (-17.48 - -0.41)	-8.22 (-18.93 - 2.48)	-10.60* (-18.64 - -2.57)	-9.74* (-18.25 - -1.23)	-9.97* (-19.59 - -0.35)	-7.68 (-16.4 - 1.03)
**Number of hospital days per patient-year**									
3 months of dialysis initiation	-0.72*** (-1.06 - -0.39)	-0.73*** (-1.08 - -0.39)	-0.11*** (-0.15 - -0.07)	-0.74*** (-1.0 - -0.38)	-0.90*** (-1.33 - -0.49)	-0.80*** (-1.13 - -0.47)	-0.72*** (-1.10 - -0.35)	-0.63** (-1.0 - -0.23)	-0.59** (-0.98 - -0.21)
**Secondary Outcomes**									
**Dual Medicare and Medicaid coverage**	2.75** (0.82 - 4.68)	2.58** (0.88 - 4.28)		2.57** (0.89 - 4.25)	2.81** (0.82 - 4.81)	2.28* (0.44 - 4.13)	2.34* (0.50 - 4.17)	2.58** (0.72 - 4.44)	2.75** (1.14 - 4.37)
**Receipt of Pre-Dialysis Nephrology Care**	0.98 (-0.13 - 2.08)	0.93 (-0.08 - 1.95)		0.91 (-0.12 - 1.94)	0.58 (-0.42 - 1.59)	0.90 (-0.09 - 1.90)	0.79 (-0.28 - 1.85)	0.77 (-0.36 - 1.90)	0.703 (-0.236 - 1.64)
**Arteriovenous fistula or graft present**	1.50* (0.18 - 2.81)	1.65* (0.31 - 3.00)		1.64* (0.27 - 3.01)	1.67* (0.09 - 3.25)	1.72* (0.32 - 3.13)	1.44* (0.12 - 2.76)	1.52* (0.08 - 2.96)	1.35 (-0.0292 - 2.74)
**Home Dialysis**	-0.10 (-1.74 - 1.53)	0.05 (-1.44 - 1.54)		0.08 (-1.44 - 1.60)	0.50 (-1.55 - 2.54)	0.66 (-0.51 - 1.82)	0.48 (-0.70 - 1.66)	-0.10 (-1.74 - 1.53)	0.0 (-1.44 - 1.46)
**Hemodialysis**	0.16	-0.03		-0.07	-0.45	-0.66	-0.48	0.10	0.16

© 2022 Nguyen KH et al. *JAMA Health Forum.*

	Unadjusted	Main Model	Poisson Model	No Lab Values	Exclude Missing Lab Values	Missing Indicator for Labs	Includes Original Reason for Medicare Entitlement	Exclude Late Expanders	Include Early Expanders
	(-1.33 - 1.65)	(-1.37 - 1.31)		(-1.45 - 1.32)	(-2.34 - 1.45)	(-1.74 - 0.42)	(-1.55 - 0.59)	(-1.35 - 1.55)	(-1.33 - 1.65)
**Peritoneal Dialysis**	-0.16 (-1.65 - 1.33)	0.03 (-1.31 - 1.37)		0.07 (-1.32 - 1.45)	0.45 (-1.45 - 2.34)	0.66 (-0.42 - 1.74)	0.48 (-0.59 - 1.55)	-0.10 (-1.55 - 1.35)	0.07 (-1.32 - 1.45)
**Cardiac Hospitalizations**									
**Hospital Admissions per 100 Patient-Years**									
3 months of dialysis initiation	-0.49 (-1.58 - 0.61)	-0.58 (-1.63 - 0.46)		-0.56 (-1.60 - 0.49)	-0.41 (-1.70 - 0.88)	-0.66 (-1.70 - 0.38)	-0.58 (-1.64 - 0.47)	-0.37 (-1.46 - 0.72)	-0.203 (-1.32 - 0.910)
6 months of dialysis initiation	-1.11 (-2.76 - 0.54)	-1.22 (-2.81 - 0.36)		-1.17 (-2.77 - 0.42)	-0.48 (-2.33 - 1.37)	-1.36 (-2.94 - 0.21)	-1.24 (-2.84 - 0.36)	-1.11 (-2.76 - 0.54)	-0.49 (-1.58 - 0.61)
12 months of dialysis initiation	-1.66 (-4.55 - 1.24)	-1.80 (-4.66 - 1.07)		-1.70 (-4.57 - 1.17)	-0.91 (-4.53 - 2.71)	-2.07 (-4.86 - 0.73)	-1.88 (-4.75 - 1.00)	-1.73 (-5.00 - 1.54)	-0.986 (-3.99 - 2.02)
**Number of hospital days per patient-year**									
3 months of dialysis initiation	-0.11 (-0.24 - 0.01)	-0.13* (-0.24 - - 0.01)	-0.08** (-0.14 - - 0.03)	-0.13* (-0.24 - - 0.01)	-0.11 (-0.24 - 0.02)	-0.14* (-0.25 - - 0.03)	-0.12* (-0.24 - - 0.01)	-0.08 (-0.19 - 0.03)	-0.08 (-0.23 - 0.07)
6 months of dialysis initiation	-0.15 (-0.33 - 0.03)	-0.16 (-0.33 - 0.01)	-0.07* (-0.13 - - 0.01)	-0.16 (-0.33 - 0.01)	-0.11 (-0.28 - 0.06)	-0.18* (-0.34 - - 0.01)	-0.16 (-0.32 - 0.01)	-0.10 (-0.27 - 0.06)	-0.09 (-0.31 - 0.13)
12 months of dialysis initiation	-0.16 (-0.40 - 0.08)	-0.18 (-0.41 - 0.05)	-0.06 (-0.11 - 0.00)	-0.17 (-0.40 - 0.05)	-0.13 (-0.42 - 0.16)	-0.20 (-0.42 - 0.01)	-0.18 (-0.40 - 0.05)	-0.13 (-0.37 - 0.11)	-0.09 (-0.36 - 0.17)
**Infection-Related Hospitalizations**									
**Hospital Admissions per 100 Patient-Years**									
3 months of dialysis initiation	-1.55*** (-2.40 - -	-1.55*** (-2.41 - -		-1.55*** (-2.42 - -	-1.68** (-2.89 - -	-1.64*** (-2.46 - -	-1.54*** (-2.40 - -	-1.60** (-2.53 - -0.67)	-1.39** (-2.26 - -

© 2022 Nguyen KH et al. *JAMA Health Forum.*

	Unadjusted	Main Model	Poisson Model	No Lab Values	Exclude Missing Lab Values	Missing Indicator for Labs	Includes Original Reason for Medicare Entitlement	Exclude Late Expanders	Include Early Expanders
	0.69)	0.68)		0.69)	0.48)	0.83)	0.67)		0.521)
6 months of dialysis initiation	-1.81** (-3.04 - -0.57)	-1.76** (-3.06 - -0.46)		-1.77** (-3.07 - -0.46)	-2.04** (-3.53 - -0.54)	-1.98** (-3.21 - -0.75)	-1.77* (-3.12 - -0.43)	-1.78* (-3.23 - -0.33)	-1.57* (-2.83 - -0.305)
12 months of dialysis initiation	-3.31** (-5.36 - -1.26)	-3.23** (-5.41 - -1.06)		-3.24** (-5.41 - -1.07)	-3.91** (-6.39 - -1.42)	-3.60** (-5.70 - -1.50)	-3.31** (-5.54 - -1.09)	-3.26* (-5.75 - -0.77)	-2.94** (-5.03 - -0.857)
**Number of hospital days per patient-year**									
3 months of dialysis initiation	-0.23*** (-0.35 - -0.10)	-0.22*** (-0.34 - -0.09)	-0.11*** (-0.17 - -0.06)	-0.22*** (-0.34 - -0.10)	-0.33*** (-0.48 - -0.18)	-0.23*** (-0.34 - -0.12)	-0.21*** (-0.33 - -0.09)	-0.20** (-0.34 - -0.07)	-0.17* (-0.30 - -0.04)
6 months of dialysis initiation	-0.23* (-0.42 - -0.04)	-0.22* (-0.40 - -0.04)	-0.07** (-0.12 - -0.02)	-0.22* (-0.41 - -0.03)	-0.37*** (-0.56 - -0.18)	-0.24** (-0.41 - -0.08)	-0.21* (-0.40 - -0.02)	-0.19 (-0.38 - 0.01)	-0.19* (-0.36 - -0.01)
12 months of dialysis initiation	-0.33* (-0.66 - -0.01)	-0.31 (-0.63 - 0.01)	-0.06* (-0.12 - -0.01)	-0.31 (-0.64 - 0.01)	-0.59*** (-0.93 - -0.26)	-0.35* (-0.65 - -0.05)	-0.30 (-0.63 - 0.03)	-0.26 (-0.63 - 0.11)	-0.25 (-0.55 - 0.05)

*p<0.05, **p<0.01, *** p<0.001. Robust 95% confidence intervals in parentheses. All estimates are the regression coefficients of an interaction term between an indicator variable for whether the patient resided in a Medicaid expansion state and time (Expansion x post).

© 2022 Nguyen KH et al. *JAMA Health Forum.*

eTable 6. Postperiod Event Study Specification

	Pre-Expansion	1-Year Post	2-Years Post	3-Years Post	4-Years Post	5-Years Post
**Primary Outcomes**						
**All-Cause Hospitalizations**						
**Hospital admissions per 100 patient-years**						
3 months of dialysis initiation	Ref	-3.08 (-6.65, 0.48)	-5.81 (-9.45, -2.17)**	-6.80 (-10.76, -2.85)**	-3.94 (-6.61, -1.28)**	-3.82 (-7.55, -0.10)*
6 months of dialysis initiation	Ref	-3.40 (-9.49, 2.69)	-7.25 (-13.63, -0.88)*	-9.92 (-17.17, -2.68)**	-5.10 (-10.3, 0.10)	-4.09 (-9.89, 1.71)
12 months of dialysis initiation	Ref	-5.61 (-16.91, 5.69)	-10.41 (-22.41, 1.60)	-14.27 (-25.95, -2.59)*	-9.54 (-18.46, -0.61)*	-9.98 (-21.28, 1.33)
**Number of hospital days per patient-year**						
3 months of dialysis initiation	Ref	-0.54 (-0.92, -0.16)**	-0.69 (-1.22, -0.17)*	-0.81 (-1.32, -0.29)**	-0.54 (-1.13, 0.05)	-0.57 (-1.18, 0.49)
**Secondary Outcomes**						
**Dual Medicare and Medicaid coverage**	Ref	2.34 (0.16, 4.52)*	3.51 (0.07, 6.94)*	1.49 (-0.52, 3.51)	2.64 (0.92, 4.37)**	2.91 (0.07, 5.74)*
**Receipt of Pre-Dialysis Nephrology Care**	Ref	-0.19 (-1.60, 1.23)	2.69 (1.16, 4.22)**	0.73 (-0.74, 2.20)	0.91 (-0.94, 2.76)	-0.35 (-1.98, 1.28)
**Arteriovenous fistula or graft present**	Ref	-0.07 (-1.59, 1.44)	0.81 (-0.83, 2.45)	2.23 (-0.0, 4.48)	2.74 (0.68, 4.80)*	1.95 (-0.05, 3.95)
**Home Dialysis**	Ref	-1.50 (-2.90, -0.11)*	0.38 (-1.39, 2.15)	0.26 (-2.18, 2.70)	0.37 (-1.59, 2.34)	-0.07 (-2.07, 1.93)
**Peritoneal Dialysis at Initiation**	Ref	-1.40 (-2.74, -0.05)*	0.66 (-1.21, 2.53)	0.26 (-1.98, 2.49)	0.27 (-1.44, 1.99)	-0.31 (-2.22, 1.59)
**Hemodialysis at Initiation**	Ref	1.40 (0.05, 2.74)*	-0.66 (-2.53, 1.21)	-0.26 (-2.49, 1.98)	-0.27 (-1.99, 1.44)	0.31 (-1.59, 2.22)
**Cardiac Hospitalizations**						
**Hospital Admissions per 100 Patient-Years**						
3 months of dialysis initiation	Ref	0.61 (-0.80, 0.20)	-0.17 (-0.01, 0.01)	-1.72 (-3.45, 0.01)	-0.60 (-1.93, 0.72)	0.04 (-1.42, 1.50)

© 2022 Nguyen KH et al. *JAMA Health Forum.*

	Pre-Expansion	1-Year Post	2-Years Post	3-Years Post	4-Years Post	5-Years Post
6 months of dialysis initiation	Ref	0.0 (-1.93, 1.94)	-1.01 (-2.80, 0.77)	-2.42 (-4.47, -0.37)*	-1.09 (-3.59, 1.41)	-0.33 (-3.05, 2.38)
12 months of dialysis initiation	Ref	0.0 (-3.55, 3.55)	-0.91 (-4.00, 2.18)	-3.86 (-7.80, 0.08)	-2.35 (-6.43, 1.74)	-1.56 (-6.30, 3.18)
**Number of hospital days per patient-year**						
3 months of dialysis initiation	Ref	-0.04 (-0.18, 0.11)	0.01 (-0.14, 0.16)	-0.14 (-0.33, 0.06)	-0.20 (-0.42, 0.02)	-0.06 (-0.23, 0.12)
6 months of dialysis initiation	Ref	-0.02 (-0.21, 0.17)	-0.01 (-0.20, 0.19)	-0.19 (-0.45, 0.06)	-0.23 (-0.56, 0.10)	-0.08 (-0.33, 0.18)
12 months of dialysis initiation	Ref	-0.09 (-0.34, 0.16)	0.02 (-0.23, 0.28)	-0.23 (-0.57, 0.10)	-0.29 (-0.71, -0.14)	-0.08 (-0.43, 0.28)
**Infection-Related Hospitalizations**						
**Hospital Admissions per 100 Patient-Years**						
3 months of dialysis initiation	Ref	0.67 (-2.67, 1.32)	-1.91 (-0.329, -0.05)**	-2.24 (-3.66, -0.82)**	-1.72 (-3.07, -0.38)*	-1.45 (-2.68, -0.22)*
6 months of dialysis initiation	Ref	0.15 (-2.34, 2.63)	-2.30 (-4.43, -0.18)*	-3.26 (-5.55, -0.97)**	-2.35 (-4.32, -0.38)*	-1.10 (-3.49, 1.28)
12 months of dialysis initiation	Ref	-0.82 (-4.39, 2.75)	-3.91 (-7.68, -0.13)*	-5.16 (-8.74, -1.58)**	-3.46 (-6.51, -0.41)*	-2.93 (-6.53, 0.66)
**Number of hospital days per patient-year**						
3 months of dialysis initiation	Ref	-0.02 (-0.30, 0.27)	0.26 (-0.45, -0.07)**	-0.38 (-0.56, -0.19)***	-0.21 (-0.41, 0.0)	-0.15 (-0.37, -0.07)
6 months of dialysis initiation	Ref	0.02 (-0.34,0.39)	-0.21 (-0.52, 0.10)	-0.44 (-0.74, -0.15)**	-0.21 (-0.51, -0.10)	-0.09 (-0.50, 0.31)
12 months of dialysis initiation	Ref	0.10 (-0.43, 0.63)	-0.39 (-0.88, 0.11)	-0.43 (-0.91, 0.04)	-0.22 (-0.73, 0.28)	-0.36 (-0.99, 0.27)

© 2022 Nguyen KH et al. *JAMA Health Forum.*

eTable 7. Differential Effects by Area-Level Poverty

	Less than 20% Population Living Below Poverty	20% or More of Population Living Below Poverty	Three-Way-Interaction (95% CI)
**Dual Medicare and Medicaid coverage**	2.77	2.49	0.27 (-1.93, 1.38)
**Receipt of Pre-Dialysis Nephrology Care**	0.89	0.99	0.10 (-1.63, 1.83)
**Arteriovenous Fistula or Graft**	1.55	1.50	-0.05 (-2.41, 2.31)
**Home Dialysis**	0.33	-0.40	-0.73, (-2.27, 0.82)
**Hospital admissions per 100 Patient-Years**			
3 months of dialysis initiation	-3.86	-4.77	0.91 (-3.64, 1.82)
6 months of dialysis initiation	-6.21	-5.72	0.49 (-5.03, 6.02)
12 months of dialysis initiation	-11.33	-7.84	3.49 (-5.26, 12.23)
**Number of Hospital Days per Patient-Year**			
3 months of dialysis initiation	-0.55	-0.91	-0.36 (-0.80, 0.08)
**Cardiac Hospitalizations**			
**Hospital Admissions per 100 Patient-Years**			
3 months of dialysis initiation	-0.23	-1.08	-0.085 (-2.37, 0.66)
6 months of dialysis initiation	-1.04	-1.69	-0.65 (-2.91, 1.60)
12 months of dialysis initiation	-2.76	-1.29	-1.46 (-2.02, 4.95)
**Number of Hospital Days per Patient-Year**			
3 months of dialysis initiation	-0.07	-0.17	-0.10, (-0.29, 0.09)
6 months of dialysis initiation	-0.11	-0.20	-0.09 (-0.38, 0.20)
12 months of dialysis initiation	-0.20	-0.15	0.05 (-0.34, 0.43)
**Infection-Related Hospitalizations**			
**Hospital Admissions per 100 Patient-Years**			
3 months of dialysis initiation	-1.23	-1.69	0.47 (-1.97, 1.04)
6 months of dialysis initiation	-2.13	-1.36	0.77 (-1.13, 2.67)
12 months of dialysis initiation	-3.72	-2.80	0.92 (-1.56, 3.40)
**Number of Hospital Days per Patient-Year**			
3 months of dialysis initiation	-0.11	-0.31	-0.19 (-0.42, 0.04)
6 months of dialysis initiation	-0.13	-0.31	-0.18 (-0.46, 0.11)
12 months of dialysis initiation	-0.30	-0.33	-0.03 (-0.49, 0.43)

Note. Estimates in “Less than 20% population living below poverty” reflect changes over time between expansion and non-expansion states among patients residing in a Census tract where less than 20% of the population lives below poverty. Estimates in “20% or more of population living below poverty” reflect changes over time between expansion and non-expansion states among patients residing in a Census tract where 20% or more of the population lives below poverty. A state’s post-period is defined by its own implementation date, which was January 1, 2014 for most expansion states. Models also adjust for age, sex, race/ethnicity, primary cause of kidney failure, comorbid conditions, being a current smoker, alcohol

dependence, and hemoglobin and serum albumin at dialysis initiation. Estimates in "three-way-interaction" is the coefficient for a three-way-interaction between indicators for Medicaid expansion, the post period, residing in a Census tract where more than 20% of the population lives below poverty (Expansion x post x poverty).

© 2022 Nguyen KH et al. *JAMA Health Forum.*

eTable 8. Differential Effects Between White and Black Patients

	White	Black	Three-Way-Interaction (95% CI)
**Dual Medicare and Medicaid coverage**	1.81	1.69	-0.12 (-2.41, 2.17)
**Receipt of Pre-Dialysis Nephrology Care**	1.10	1.43	0.33 (-1.31, 1.97)
**Arteriovenous Fistula or Graft**	1.24	1.61	0.37 (-1.59, 2.33)
**Home Dialysis**	0.05	-0.20	-0.26 (-1.95, 1.44)
**Hospital admissions per 100 Patient-Years**			
3 months of dialysis initiation	-5.85	-3.75	2.10 (-1.63, 5.82)
6 months of dialysis initiation	-8.14	-4.89	3.24 (-2.84, 9.33)
12 months of dialysis initiation	-12.58	-8.88	3.70 (-6.98, 14.38)
**Number of Hospital Days per Patient-Year**			
3 months of dialysis initiation	-0.80	-0.57	0.23 (-0.33, 0.78)
**Cardiac Hospitalizations**			
**Hospital Admissions per 100 Patient-Years**			
3 months of dialysis initiation	-0.71	-1.07	-0.36 (-1.92, 1.21)
6 months of dialysis initiation	-1.28	-2.27	-0.99 (-3.38, 1.40)
12 months of dialysis initiation	-2.60	-2.77	-0.18 (-4.01, 3.66)
**Number of Hospital Days per Patient-Year**			
3 months of dialysis initiation	-0.12	-0.13	-0.01 (-0.25, 0.22)
6 months of dialysis initiation	-0.15	-0.16	-0.01 (-0.28, 0.27)
12 months of dialysis initiation	-0.21	-0.16	0.05 (-0.27, 0.37)
**Infection-Related Hospitalizations**			
**Hospital Admissions per 100 Patient-Years**			
3 months of dialysis initiation	-1.68	-2.33	-0.65 (-2.21, 0.90)
6 months of dialysis initiation	-1.97	-2.17	-0.20 (-2.44, 2.03)
12 months of dialysis initiation	-3.37	-5.25	-1.87 (-5.32, 1.58)
**Number of Hospital Days per Patient-Year**			
3 months of dialysis initiation	-0.16	-0.29	-0.14 (-0.39, 0.12)
6 months of dialysis initiation	-0.18	-0.25	-0.07 (-0.48, 0.33)
12 months of dialysis initiation	-0.28	-0.53	-0.25 (-0.73, 0.23)

Note. Estimates reflect difference between expansion and non-expansion states. Models include indicators for Medicaid expansion, the post period, age group, and their interaction (Expansion x post x race/ethnicity). A state's post-period is defined by its own implementation date, which was January 1, 2014 for most expansion states. Models also adjust for sex, race/ethnicity, primary cause of kidney failure, comorbid conditions, being a current smoker, alcohol dependence, hemoglobin and serum

© 2022 Nguyen KH et al. *JAMA Health Forum.*

albumin at dialysis initiation, and area-level poverty. Estimates in "three-way-interaction" is the coefficient for a three-way-interaction between indicators for Medicaid expansion, the post period, and race/ethnicity (Expansion x post x race/ethnicity).

© 2022 Nguyen KH et al. *JAMA Health Forum.*

eTable 9. Differential Effects Between White and Hispanic or Latino Patients

	White	Hispanic/ Latino	Three-Way-Interaction (95% CI)
**Dual Medicare and Medicaid coverage**	1.81	7.40	5.59 (3.32, 7.86)***
**Receipt of Pre-Dialysis Nephrology Care**	1.10	0.80	-0.30 (-2.80, 2.20)
**Arteriovenous Fistula or Graft**	1.24	3.58	2.35 (0.32 4.38)*
**Home Dialysis**	0.05	0.44	0.39 (-1.20, 1.98)
**Hospital admissions per 100 Patient-Years**			
3 months of dialysis initiation	-5.85	-2.29	3.56 (-0.58, 7.70)
6 months of dialysis initiation	-8.14	-4.58	3.56 (-2.12, 9.24)
12 months of dialysis initiation	-12.58	-4.92	7.65 (-3.08, 18.39)
**Number of Hospital Days per Patient-Year**			
3 months of dialysis initiation	-0.80	-0.95	-0.16 (-0.69, 0.38)
**Cardiac Hospitalizations**			
**Hospital Admissions per 100 Patient-Years**			
3 months of dialysis initiation	-0.71	0.49	1.20 (-0.48, 2.89)
6 months of dialysis initiation	-1.28	0.35	1.63 (-0.83, 4.09)
12 months of dialysis initiation	-2.60	1.00	3.59 (-0.52, 7.70)
**Number of Hospital Days per Patient-Year**			
3 months of dialysis initiation	-0.12	-0.22	-0.11 (-0.29, 0.07)
6 months of dialysis initiation	-0.15	-0.24	-0.09 (-0.34, 0.16)
12 months of dialysis initiation	-0.21	-0.25	-0.04 (-0.37, 0.29)
**Infection-Related Hospitalizations**			
**Hospital Admissions per 100 Patient-Years**			
3 months of dialysis initiation	-1.68	-1.07	-0.65 (-2.21, 0.90)
6 months of dialysis initiation	-1.97	-2.09	-0.13 (-2.51, 2.26)
12 months of dialysis initiation	-3.37	-0.92	2.45 (-1.66, 6.57)
**Number of Hospital Days per Patient-Year**			
3 months of dialysis initiation	-0.16	-0.23	-0.07 (-0.29, 0.14)
6 months of dialysis initiation	-0.18	-0.27	-0.10 (-0.40, 0.21)
12 months of dialysis initiation	-0.28	-0.09	0.20 (-0.25, 0.64)

Note. Estimates reflect difference between expansion and non-expansion states. Models include indicators for Medicaid expansion, the post period, age group, and their interaction (Expansion x post x race/ethnicity). A state's post-period is defined by its own implementation date, which was January 1, 2014 for most expansion states. Models also adjust for sex, race/ethnicity, primary cause of kidney failure, comorbid conditions, being a current smoker, alcohol dependence, hemoglobin and serum albumin at dialysis initiation, and area-level poverty. Estimates in "three-way-interaction" is the coefficient for a three-way-interaction between indicators for Medicaid expansion, the post period, and race/ethnicity (Expansion x post x race/ethnicity).

eTable 10. Differential Effects Between White and Asian American Patients

	White	Asian American	Three-Way-Interaction (95% CI)
**Dual Medicare and Medicaid coverage**	1.81	0.94	0.12 (-2.41, 2.17)
**Receipt of Pre-Dialysis Nephrology Care**	1.10	2.30	1.20 (-5.36, 7.76)
**Arteriovenous Fistula or Graft**	1.24	1.91	0.67 (-8.75, 10.10)
**Home Dialysis**	0.05	2.21	2.16 (-4.21, 8.52)
**Hospital admissions per 100 Patient-Years**			
3 months of dialysis initiation	-5.85	2.46	8.31 (0.06, 16.56)*
6 months of dialysis initiation	-8.14	-1.15	6.98 (-5.57, 20.63)
12 months of dialysis initiation	-12.58	-4.47	8.11 (-11.31, 27.53)
**Number of Hospital Days per Patient-Year**			
3 months of dialysis initiation	-0.80	-0.27	0.53 (-0.94, 1.99)
**Cardiac Hospitalizations**			
**Hospital Admissions per 100 Patient-Years**			
3 months of dialysis initiation	-0.71	0.53	1.24 (-4.55, 7.04)
6 months of dialysis initiation	-1.28	-3.31	-2.03 (-8.58, 4.53)
12 months of dialysis initiation	-2.60	0.86	3.46 (-4.11, 12.03)
**Number of Hospital Days per Patient-Year**			
3 months of dialysis initiation	-0.12	-0.05	-0.07 (-0.61, 0.75)
6 months of dialysis initiation	-0.15	-0.46	-0.31 (-1.02, 0.40)
12 months of dialysis initiation	-0.21	0.01	0.22 (-0.62, 1.06)
**Infection-Related Hospitalizations**			
**Hospital Admissions per 100 Patient-Years**			
3 months of dialysis initiation	-1.68	0.49	-0.61 (-1.17, 2.39)
6 months of dialysis initiation	-1.97	-1.56	0.40 (-4.44, 5.24)
12 months of dialysis initiation	-3.37	-4.11	-0.74 (-11.18, 9.70)
**Number of Hospital Days per Patient-Year**			
3 months of dialysis initiation	-0.16	0.06	0.21 (-0.43, 0.85)
6 months of dialysis initiation	-0.18	-0.23	-0.05 (-0.76, 0.67)
12 months of dialysis initiation	-0.28	-0.47	-0.18 (-1.54, 1.17)

Note. Estimates reflect difference between expansion and non-expansion states. Models include indicators for Medicaid expansion, the post period, age group, and their interaction (Expansion x post x race/ethnicity). A state's post-period is defined by its own implementation date, which was January 1, 2014 for most expansion states. Models also adjust for sex, race/ethnicity, primary cause of kidney failure, comorbid conditions, being a current smoker, alcohol dependence, hemoglobin and serum albumin at dialysis initiation, and area-level poverty. Estimates in "three-way-interaction" is the coefficient for a three-way-interaction between indicators for Medicaid expansion, the post period, and race/ethnicity (Expansion x post x race/ethnicity).

© 2022 Nguyen KH et al. *JAMA Health Forum.*

eTable 11. Differential Effects Between White and Other Race Patients

	White	Other Race	Three-Way-Interaction (95% CI)
**Dual Medicare and Medicaid coverage**	1.81	6.22	4.41 (-4.30, 13.13)
**Receipt of Pre-Dialysis Nephrology Care**	1.10	-0.86	-1.96 (-6.29, 2.37)
**Arteriovenous Fistula or Graft**	1.24	-0.77	-2.01 (-8.06, 4.04)
**Home Dialysis**	0.05	-2.07	-2.12 (-9.92, 5.67)
**Hospital admissions per 100 Patient-Years**			
3 months of dialysis initiation	-5.85	-4.06	1.79 (-13.16, 16.73)
6 months of dialysis initiation	-8.14	2.94	11.07 (-12.63, 34.77)
12 months of dialysis initiation	-12.58	-0.11	12.47 (-22.43, 47.37)
**Number of Hospital Days per Patient-Year**			
3 months of dialysis initiation	-0.80	-1.50	-0.70 (-2.42, 1.01)
**Cardiac Hospitalizations**			
**Hospital Admissions per 100 Patient-Years**			
3 months of dialysis initiation	-0.71	-0.53	0.19 (-6.10, 6.47)
6 months of dialysis initiation	-1.28	-1.37	-0.09 (-9.62, 9.43)
12 months of dialysis initiation	-2.60	-2.16	0.44 (-12.59, 13.46)
**Number of Hospital Days per Patient-Year**			
3 months of dialysis initiation	-0.12	-0.26	-0.15 (-0.86, 0.57)
6 months of dialysis initiation	-0.15	-0.49	-0.34 (1.01, 0.34)
12 months of dialysis initiation	-0.21	-0.60	-0.39 (-1.23, 0.45)
**Infection-Related Hospitalizations**			
**Hospital Admissions per 100 Patient-Years**			
3 months of dialysis initiation	-1.68	0.86	2.54 (-3.77, 8.86)
6 months of dialysis initiation	-1.97	0.15	2.12 (-8.28, 12.52)
12 months of dialysis initiation	-3.37	-4.43	-1.06 (-16.07, 13.95)
**Number of Hospital Days per Patient-Year**			
3 months of dialysis initiation	-0.16	-1.04	-0.89 (-1.94, 17.0)
6 months of dialysis initiation	-0.18	-1.38	-1.21 (-2.53, 11.95)
12 months of dialysis initiation	-0.28	-1.50	-1.21 (-3.11, 0.68)

Note. Estimates reflect difference between expansion and non-expansion states. Models include indicators for Medicaid expansion, the post period, age group, and their interaction (Expansion x post x race/ethnicity). A state's post-period is defined by its own implementation date, which was January 1, 2014 for most expansion states. Models also adjust for sex, race/ethnicity, primary cause of kidney failure, comorbid conditions, being a current smoker, alcohol dependence, hemoglobin and serum albumin at dialysis initiation, and area-level poverty. Estimates in "three-way-interaction" is the coefficient for a three-way-interaction between indicators for Medicaid expansion, the post period, and race/ethnicity (Expansion x post x race/ethnicity).

© 2022 Nguyen KH et al. *JAMA Health Forum.*

eTable 12. Differential Effects Between Patients Aged 18 to 34 vs 35 to 44 Years

	18-34	35-44	Three-Way-Interaction (95% CI)
**Dual Medicare and Medicaid coverage**	-0.49	1.37	1.86 (-3.00, 6.72)
**Receipt of Pre-Dialysis Nephrology Care**	-1.10	0.75	1.85 (-1.83, 5.54)
**Arteriovenous Fistula or Graft**	0.32	0.48	0.17 (-4.30, 4.64)
**Home Dialysis**	-2.19	0.00	2.19 (-2.06, 6.44)
**Hospital admissions per 100 Patient-Years**			
3 months of dialysis initiation	-0.59	-3.83	-3.24 (-12.07, 5.59)
6 months of dialysis initiation	-1.17	-7.42	-4.81 (-18.38, 8.75)
12 months of dialysis initiation	-2.29	-9.10	-8.90 (-30.8, 13.03)
**Number of Hospital Days per Patient-Year**			
3 months of dialysis initiation	0.15	-0.28	-0.43 (1.40, 0.54)
**Cardiac Hospitalizations**			
**Hospital Admissions per 100 Patient-Years**			
3 months of dialysis initiation	-0.94	0.45	1.39 (-1.81, 4.60)
6 months of dialysis initiation	0.18	-0.26	-0.45 (-6.61, 5.72)
12 months of dialysis initiation	2.44	0.09	-2.34 (-12.14, 7.44)
**Number of Hospital Days per Patient-Year**			
3 months of dialysis initiation	-0.06	0.15	0.21 (-0.16, 0.58)
6 months of dialysis initiation	0.05	0.09	0.03 (-0.50, 0.57)
12 months of dialysis initiation	0.33	0.15	-0.18 (-0.90, 0.53)
**Infection-Related Hospitalizations**			
**Hospital Admissions per 100 Patient-Years**			
3 months of dialysis initiation	-2.35	-0.65	1.71 (-1.51, 4.92)
6 months of dialysis initiation	-2.36	-2.30	0.06 (-6.17, 6.29)
12 months of dialysis initiation	-6.49	-0.95	5.54 (-4.07, 15.13)
**Number of Hospital Days per Patient-Year**			
3 months of dialysis initiation	-0.01	0.16	0.17 (-0.31, 0.66)
6 months of dialysis initiation	-0.18	0.27	-0.45 (-0.33, 1.23)
12 months of dialysis initiation	-0.40	0.30	0.70 (-0.29, 1.69)

Note. Estimates reflect difference between expansion and non-expansion states. Models include indicators for Medicaid expansion, the post period, age group, and their interaction (Expansion x post x age). A state's post-period is defined by its own implementation date, which was January 1, 2014 for most expansion states. Models also adjust for sex, race/ethnicity, primary cause of kidney failure, comorbid conditions, being a current smoker, alcohol dependence, hemoglobin and serum albumin at dialysis initiation, and area-level poverty. Estimates in "three-way-interaction" is the coefficient for a three-way-interaction between indicators for Medicaid expansion, the post period, and age category (Expansion x post x race/ethnicity).

© 2022 Nguyen KH et al. *JAMA Health Forum.*

eTable 13. Differential Effects Between Patients Aged 18 to 34 vs 45 to 54 Years

	18-34	45-54	Three-Way-Interaction (95% CI)
**Dual Medicare and Medicaid coverage**	-0.49	2.11	2.60 (-1.82, 7.02)
**Receipt of Pre-Dialysis Nephrology Care**	-1.10	0.72	1.82 (-1.79, 5.43)
**Arteriovenous Fistula or Graft**	0.32	1.28	0.96 (-3.09, 5.02)
**Home Dialysis**	-2.19	-0.25	1.94 (-2.26, 6.14)
**Hospital admissions per 100 Patient-Years**			
3 months of dialysis initiation	-0.59	-5.13	-4.54 (-12.08, 3.00)
6 months of dialysis initiation	-1.17	-5.99	-4.81 (-18.38, 8.75)
12 months of dialysis initiation	-2.29	-7.05	-4.77 (-29.82, 20.28)
**Number of Hospital Days per Patient-Year**			
3 months of dialysis initiation	0.15	-0.71	-0.86 (-1.90, 0.18)
**Cardiac Hospitalizations**			
**Hospital Admissions per 100 Patient-Years**			
3 months of dialysis initiation	-0.94	-1.28	-0.34 (-3.37, 2.69)
6 months of dialysis initiation	0.18	-1.51	-1.69 (-6.46, 3.09)
12 months of dialysis initiation	2.44	-2.56	-4.99 (-12.91, 2.92)
**Number of Hospital Days per Patient-Year**			
3 months of dialysis initiation	-0.06	-0.12	-0.06 (-0.31, 0.19)
6 months of dialysis initiation	0.05	-0.18	-0.23 (-0.57, 0.11)
12 months of dialysis initiation	0.33	-0.19	-0.52 (-1.03, -0.003)*
**Infection-Related Hospitalizations**			
**Hospital Admissions per 100 Patient-Years**			
3 months of dialysis initiation	-2.35	-2.03	0.32 (-3.10, 3.75)
6 months of dialysis initiation	-2.36	-2.24	0.12 (-5.61, 5.85)
12 months of dialysis initiation	-6.49	-3.14	0.90 (-4.23, 6.02)
**Number of Hospital Days per Patient-Year**			
3 months of dialysis initiation	-0.01	-0.21	-0.20 (-0.67, 0.27)
6 months of dialysis initiation	-0.18	-0.33	-0.15 (-0.94, 0.64)
12 months of dialysis initiation	-0.40	-0.43	0.04 (-0.76, 0.85)

Note. Estimates reflect difference between expansion and non-expansion states. Models include indicators for Medicaid expansion, the post period, age group, and their interaction (Expansion x post x age). A state's post-period is defined by its own implementation date, which was January 1, 2014 for most expansion states. Models also adjust for sex, race/ethnicity, primary cause of kidney failure, comorbid conditions, being a current smoker, alcohol dependence, hemoglobin and serum albumin at dialysis initiation, and area-level poverty. Estimates in "three-way-interaction" is the coefficient for a three-way-interaction between indicators for Medicaid expansion, the post period, and age category (Expansion x post x age).

© 2022 Nguyen KH et al. *JAMA Health Forum.*

eTable 14. Differential Effects Between Patients Aged 18 to 34 vs 55 to 64 Years

	18-34	55-64	Three-Way-Interaction (95% CI)
**Dual Medicare and Medicaid coverage**	-0.49	3.59	4.08 (-0.09, 8.26)
**Receipt of Pre-Dialysis Nephrology Care**	-1.10	1.30	2.40 (-0.78, 5.59)
**Arteriovenous Fistula or Graft**	0.32	2.07	1.75 (-2.11, 5.61)
**Home Dialysis**	-2.19	0.33	2.52 (-1.32, 6.35)
**Hospital admissions per 100 Patient-Years**			
3 months of dialysis initiation	-0.59	-4.32	-3.74 (-11.98, 4.51)
6 months of dialysis initiation	-1.17	-6.09	-4.92 (-16.95, 7.11)
12 months of dialysis initiation	-2.29	-11.19	-8.90 (-30.84, 13.03)
**Number of Hospital Days per Patient-Year**			
3 months of dialysis initiation	0.15	-0.86	-1.00 (-2.02, 0.01)
**Cardiac Hospitalizations**			
**Hospital Admissions per 100 Patient-Years**			
3 months of dialysis initiation	-0.94	-0.36	0.57 (-2.07, 3.21)
6 months of dialysis initiation	0.18	-1.41	-1.59 (-6.00, 2.81)
12 months of dialysis initiation	2.44	-2.24	-4.68 (-12.04, 2.69)
**Number of Hospital Days per Patient-Year**			
3 months of dialysis initiation	-0.06	-0.17	-0.10 (-0.37, 0.17)
6 months of dialysis initiation	0.05	-0.20	-0.26 (-0.62, 0.11)
12 months of dialysis initiation	0.33	-0.27	-0.60 (-1.15, -0.05)*
**Infection-Related Hospitalizations**			
**Hospital Admissions per 100 Patient-Years**			
3 months of dialysis initiation	-2.35	-1.45	0.90 (-2.44, 4.24)
6 months of dialysis initiation	-2.36	-1.46	0.90 (-4.23, 6.02)
12 months of dialysis initiation	-6.49	-3.41	3.07 (-4.68, 10.83)
**Number of Hospital Days per Patient-Year**			
3 months of dialysis initiation	-0.01	-0.30	-0.29 (-0.75, 0.16)
6 months of dialysis initiation	-0.18	-0.25	-0.07 (-0.70, 0.57)
12 months of dialysis initiation	-0.40	-0.35	0.04 (-0.75, 0.85)

Note. Estimates reflect difference between expansion and non-expansion states. Models include indicators for Medicaid expansion, the post period, age group, and their interaction (Expansion x post x age). A state's post-period is defined by its own implementation date, which was January 1, 2014 for most expansion states. Models also adjust for sex, race/ethnicity, primary cause of kidney failure, comorbid conditions, being a current smoker, alcohol dependence, hemoglobin and serum albumin at dialysis initiation, and area-level poverty. Estimates in "three-way-interaction" is the coefficient for a three-way-interaction between indicators for Medicaid expansion, the post period, and age category (Expansion x post x age).

© 2022 Nguyen KH et al. *JAMA Health Forum.*

eTable 15. Differential Effects by Sex

	Male	Female	Three-Way-Interaction (95% CI)
**Dual Medicare and Medicaid coverage**	2.85	2.46	-0.39 (-2.41, 1.63)
**Receipt of Pre-Dialysis Nephrology Care**	1.43	0.25	-1.19 (-2.81, 0.44)
**Arteriovenous Fistula or Graft**	1.38	2.07	0.69 (-0.82, 2.20)
**Home Dialysis**	-0.03	-0.06	-0.02 (-1.26, 1.21)
**Hospital admissions per 100 Patient-Years**			
3 months of dialysis initiation	-4.54	-3.83	0.71 (-2.22, 3.64)
6 months of dialysis initiation	-6.40	-5.22	1.18 (-3.49, 5.86)
12 months of dialysis initiation	-9.84	-8.70	1.14 (-7.45, 9.72)
**Number of Hospital Days per Patient-Year**			
3 months of dialysis initiation	-0.74	-0.68	0.07 (-0.40, 0.53)
**Cardiac Hospitalizations**			
**Hospital Admissions per 100 Patient-Years**			
3 months of dialysis initiation	-0.75	-0.30	0.45 (-1.29, 2.19)
6 months of dialysis initiation	-2.00	-0.10	1.91 (-0.49, 4.30)
12 months of dialysis initiation	-2.92	-0.24	2.68 (-0.82, 6.19)
**Number of Hospital Days per Patient-Year**			
3 months of dialysis initiation	-0.08	-0.17	-0.10 (-0.22, 0.03)
6 months of dialysis initiation	-0.17	-0.14	0.03 (-0.13, 0.19)
12 months of dialysis initiation	-0.18	-0.16	0.03 (-0.16, 0.22)
**Infection-Related Hospitalizations**			
**Hospital Admissions per 100 Patient-Years**			
3 months of dialysis initiation	-1.29	-1.97	-0.68 (-1.76, 0.04)
6 months of dialysis initiation	-1.60	-2.12	-0.52 (-2.52, 1.48)
12 months of dialysis initiation	-3.03	-3.57	-0.54 (-3.35, 2.27)
**Number of Hospital Days per Patient-Year**			
3 months of dialysis initiation	-0.21	-0.21	0.0 (-0.24, 0.25)
6 months of dialysis initiation	-0.24	-0.18	0.05 (-0.30, 0.40)
12 months of dialysis initiation	-0.30	-0.31	-0.01 (-0.44, 0.41)

Note. Estimates reflect difference between expansion and non-expansion states. Models include indicators for Medicaid expansion, the post period, sex, and their interaction (Expansion x post x sex). A state's post-period is defined by its own implementation date, which was January 1, 2014 for most expansion states. Models also adjust for age, race/ethnicity, primary cause of kidney failure, comorbid conditions, being a current smoker, alcohol dependence, hemoglobin and serum albumin at dialysis initiation, and area-level poverty. Estimates in "three-way-interaction" is the coefficient for a three-way-interaction between indicators for Medicaid expansion, the post period, and sex (Expansion x post x sex).

© 2022 Nguyen KH et al. *JAMA Health Forum.*

eTable 16. Changes in Mortality Rates per 100 Patient-Years by State Expansion Status

	**Expansion**		**Non-Expansion**		**Adjusted Difference-in-Differences Estimate (95% Confidence Interval)**	**p-value**
**Mortality Rate per 100 Patient-Years**	Pre	Post	2010-2013	2014-2018		
3 months of dialysis initiation	4.12	3.79	3.71	3.68	-0.27 (-0.23, 0.86)	0.08
6 months of dialysis initiation	8.02	7.26	7.47	7.17	-0.48 (-0.28, 0.40)	0.07
12 months of dialysis initiation	14.26	13.22	13.52	13.15	-0.55 (-1.09, -0.01)	0.04*

*p<0.05, **p<0.01, *** p<0.001. Estimates in "Non-Expansion" and "Expansion" columns are unadjusted. Adjusted estimates are the regression coefficients of an interaction term between an indicator variable for whether the patient resided in a Medicaid expansion state and time (Expansion x post) and adjust for covariates in the Main Model, include state- and year-quarter fixed effects, and cluster standard errors at the state-level.

© 2022 Nguyen KH et al. *JAMA Health Forum.*

eReferences.

1. Status of State Medicaid Expansion Decisions. 2022. (Accessed February 14, 2022, at https://www.kff.org/medicaid/issue-brief/status-of-state-medicaid-expansion-decisions-interactive-map/.)
2. Miller S, Wherry LR. Health and Access to Care during the First 2 Years of the ACA Medicaid Expansions. N Engl J Med 2017;376:947-56.

© 2022 Nguyen KH et al. *JAMA Health Forum.*
